# Association between psychiatric disorders and intracranial aneurysms: evidence from Mendelian randomization analysis

**DOI:** 10.3389/fneur.2024.1422984

**Published:** 2024-07-26

**Authors:** Sichen Bao, Zhenqiu Xing, Shengkai He, Xiaowei Hu, Jianjing Yang, Bingqing Zhou

**Affiliations:** ^1^Department of Neurosurgery, The First Affiliated Hospital of Wenzhou Medical University, Wenzhou, Zhejiang, China; ^2^Zhejiang-US Joint Laboratory for Aging and Neurological Disease Research, The First Affiliated Hospital of Wenzhou Medical University, Wenzhou, China; ^3^Zhejiang Provincial Key Laboratory of Aging and Neurological Disorder Research, The First Affiliated Hospital of Wenzhou Medical University, Wenzhou, China; ^4^Department of Thoracic Surgery, The First Affiliated Hospital of Wenzhou Medical University, Wenzhou, Zhejiang, China

**Keywords:** intracranial aneurysms, psychiatric disorders, schizophrenia, cognitive impairment, Mendelian randomization

## Abstract

**Objective:**

Several studies have explored the relationship between intracranial aneurysms and psychiatric disorders; nevertheless, the causal connection remains ambiguous. This study aimed to evaluate the causal link between intracranial aneurysms and specific psychiatric disorders.

**Methods:**

A two-sample Mendelian randomization (MR) analysis was conducted utilizing aggregated genome-wide association study (GWAS) data from the International Stroke Genetics Association for Intracranial Aneurysms (IAs), unruptured Intracranial Aneurysm (uIA), and aneurysmal Subarachnoid Hemorrhage (aSAH). Psychiatric disorder data, encompassing Schizophrenia (SCZ), Bipolar Disorder (BD), and Panic Disorder (PD), were sourced from the Psychiatric Genomics Consortium (PGC), while Cognitive Impairment (CI) data, comprising Cognitive Function (CF) and Cognitive Performance (CP), were obtained from IEU OpenGWAS publications. Causal effects were evaluated using inverse variance weighted (IVW), MR-Egger, and weighted median methods, with the robustness of findings assessed via sensitivity analyses employing diverse methodological approaches.

**Results:**

Our MR analysis indicated no discernible causal link between intracranial aneurysm (IA) and an elevated susceptibility to psychiatric disorders. However, among individuals with genetically predisposed unruptured intracranial aneurysms (uIA), there was a modest reduction in the risk of SCZ (IVW odds ratio [OR] = 0.95, 95% confidence interval [CI] 0.92–0.98, *p* = 0.0002). Similarly, IAs also exhibited a moderate reduction in SCZ risk (OR = 0.92, 95% CI 0.86–0.99, *p* = 0.02). Nevertheless, limited evidence was found to support a causal association between intracranial aneurysms and the risk of the other three psychiatric disorders.

**Conclusion:**

Our findings furnish compelling evidence suggesting a causal influence of intracranial aneurysms on psychiatric disorders, specifically, both IAs and uIA exhibit a negative causal association with SCZ.

## Introduction

Intracranial aneurysm is characterized by an abnormal protrusion of the artery wall within the cranial cavity. The global incidence is approximately 3.2% (1), with subarachnoid hemorrhage (SAH) from aneurysmal rupture accounting for about 80% of cases (2). Aneurysm rupture ranks as the second leading cause of cerebrovascular accidents, following cerebral thrombosis and hypertensive cerebral hemorrhage. Cerebral aneurysm is associated with high rates of mortality and disability. Most intracranial aneurysms are asymptomatic before rupture, though some patients may experience headaches, transient ischemic attacks, cranial neuropathy, or seizures (3). Research shows a rising incidence of aneurysmal subarachnoid hemorrhage (aSAH) with age, particularly among women aged 55 and older (4). As the population ages, intracranial aneurysms may pose an increasing public health issue (5). The literature highlights substantial functional impairment following aSAH (6), resulting in significant societal and familial burdens (7).

Psychiatric disorders include various complex cognitive and psychological syndromes, such as cognitive impairment (CI), major depressive disorder (MDD), schizophrenia (SCZ), bipolar disorder (BD), and panic disorder (PD). These conditions are characterized by cognitive, emotional, and behavioral changes, often accompanied by distress and functional impairment. With high morbidity and mortality rates, psychiatric disorders present a significant public health challenge, imposing substantial burdens on individuals, society, and the economy (8). Numerous studies have demonstrated a bidirectional causal relationship between intracranial aneurysms (IAs) and MDD, indicating that IA patients have an elevated risk of developing MDD (9, 10). MR analysis of correlations has further validated this causal association (11). Additionally, some studies have observed the first occurrence of intracranial aneurysms presenting as SCZ or BD (12), and cognitive impairment has been identified as a lasting consequence in individuals who have experienced aneurysmal subarachnoid hemorrhage (aSAH) (13). Observational studies may be biased due to various confounding factors, complicating the establishment of a causal relationship between intracranial aneurysms and psychiatric disorders.

Mendelian randomization (MR) is an epidemiological approach that uses genetic variants closely associated with exposure as instrumental variables (IVs) to assess causal relationships between risk factors and specific diseases (14). By utilizing genetic variants as IVs and taking advantage of their consistent, random, and independent distribution during meiosis, this method effectively avoids confounding and reverse causality issues (15). This technique increases the reliability of causal inference, addresses common limitations of observational studies, and is widely used to validate their results (16). In this study, MR analysis was used to investigate the potential causal links between intracranial aneurysms (IAs), unruptured intracranial aneurysms (uIA), aneurysmal subarachnoid hemorrhage (aSAH), and psychiatric disorders such as schizophrenia (SCZ), bipolar disorder (BD), panic disorder (PD), and cognitive impairment (CI).

## Methods

### Study design

**Figure 1:**
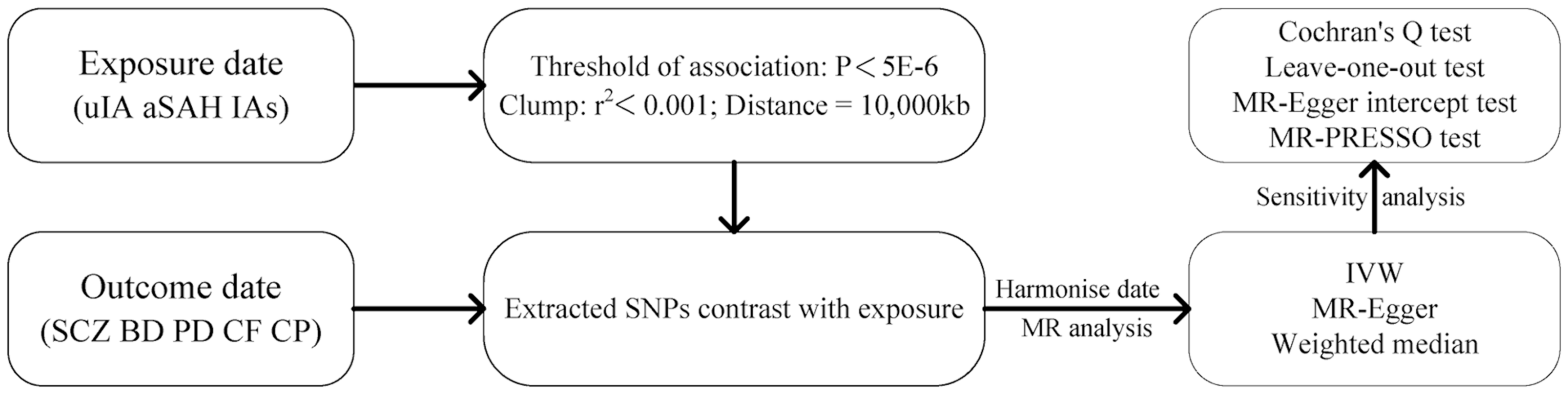
The flowchart of Mendelian randomization study design in this study. IAs, intracranial aneurysms; uIA, unruptured intracranial aneurysm; aSAH, aneurysmal subarachnoid hemorrhage; SCZ, schizophrenia; BD, bipolar disorder; PD, panic disorder; CF, cognitive function; CP, cognitive performance.

Four psychiatric disorders were chosen as outcome variables, while single nucleotide polymorphisms (SNPs) strongly associated with IAs, uIA, and SAH were selected as instrumental variables. MR analysis was employed to investigate causal relationships between exposure factors and outcome variables. To ensure result reliability, heterogeneity tests and pleiotropy analyses were conducted to mitigate potential biases. To ensure the reliability of MR (17), three key assumptions were employed: (1) the association hypothesis, wherein IVs are highly correlated with exposure factors; (2) the independence hypothesis, where IVs remain independent of any confounding factors associated with exposure and outcome variables; and (3) the exclusion hypothesis, positing that IVs solely influence outcomes via exposure factors. The research design is depicted in Figure 1.

### Date source

The data for IAs, UIA, and aSAH were acquired from the International Society for Stroke Genetics.^1^ The study performed cross-ethnic genome-wide association studies (GWAS) involving 10,754 cases and 306,882 controls of European and East Asian descent. To maintain ethnic consistency, only European samples were utilized for Mendelian randomization (MR) analysis (18).

The study included data from the Psychiatric Genomics Consortium (PGC), encompassing 130,644 patients with SCZ (comprising 53,386 cases and 77,258 controls), 413,466 patients with BD (comprising 41,917 cases and 371,549 controls), and 10,240 patients with PD (comprising 2,248 cases and 7,992 controls), as derived from partial GWAS statistics.^2^ Furthermore, two datasets from the IEU OpenGWAS database were utilized.^3^ The first dataset comprised aggregated data from 22,593 samples assessing cognitive function (ieu-b-4838), provided by the Within Family GWAS Consortium. This dataset employs cognitive function scores as a measure, where higher scores indicate better cognitive function and vice versa. The second dataset, Cognitive Performance (ebi-a-GCST006572), pooled data from 257,841 samples, all of European ancestry.

The specifics of the genetic instrumental variables utilized in the study are presented in Table 1.

**Table 1:** The GWAS data source details.

Phenotype	Data source	Consortium	Sample size	Ancestry	PMID
IAs	ISGC	Bakker et al.	79,429	European	33,199,917
uIA	ISGC	Bakker et al.	74,004	European	33,199,918
aSAH	ISGC	Bakker et al.	77,074	European	33,199,919
Schizophrenia	PGC	Trubetskoy et al.	130,644	European	35,396,580
Bipolar disorder	PGC	Mullins et al.	413,466	European	34,002,096
Panic disorder	PGC	Forstner et al.	10,240	European	31,712,720
Cognitive performance	IEU OpenGWAS	SSGAC	257,841	European	30,038,396
Cognitive function	IEU OpenGWAS	Within family GWAS consortium	22,593	European	35,534,559

IAs, intracranial aneurysms; uIA, unruptured intracranial aneurysm; aSAH, aneurysmal subarachnoid hemorrhage; ISGC, International Stroke Genetics Consortium; PGC, Psychiatric Genomics Consortium.

### Instrument variables selection

To fulfill the primary MR hypothesis asserting a robust association between single nucleotide polymorphisms (SNPs) and intracranial aneurysms (IAs), unruptured intracranial aneurysms (uIA), and aneurysmal subarachnoid hemorrhage (aSAH), we faced a scarcity of SNP-IA associations meeting the stringent genome-wide association threshold (*p* < 5.00E−8) within the aggregated GWAS dataset. Consequently, we resorted to utilizing significance levels (*p* < 5 × 10^–6^) for the identification of IVs. Subsequently, we established the linkage disequilibrium coefficient at r^2^ < 0.001 and confined the region width to 10,000 kb to minimize potential pleiotropic effects on the outcomes. Additionally, palindrome SNPs were manually removed from consideration. The SNPs that persisted after these procedures were then designated as instrumental variables. To mitigate any potential weak instrumental bias, we employed the F statistic (F = β2/se2) to evaluate the statistical power of the SNPs (19). Notably, an *F* value exceeding 10 signifies the absence of weak instrumental bias for the chosen instrumental variable.

### Mendelian randomization analysis

In this study, we employed the inverse-variance weighted (IVW), MR-Egger, and Weighted median (WM) methods to investigate the presence of a causal relationship between the exposure and outcome variables (20). The IVW method integrates Wald estimates of genetic causality for each SNP to evaluate the influence of exposure on outcomes, presupposing the validity of all chosen SNPs as instrumental variables. This method offers the most precise estimate and serves as the principal statistical approach for evaluating causal effects (21). Moreover, the weighted median method is capable of providing a dependable estimate of the causal effect, even when as much as 50% of the data utilized in the analysis stems from genetic variants that might not qualify as valid instrumental variables (22). MR-Egger permits genetic instrumental variables to exhibit pleiotropic effects but mandates that these effects remain independent of the associations between variations and exposures (23). The MR-Egger and weighted median methods were supplemented to provide a more robust and extensive assessment.

### Sensitive analysis

To ensure the reliability of the study outcomes, a comprehensive set of sensitivity analyses and quality controls were executed. The heterogeneity among genetic variants employed as instrumental variables was evaluated employing Cochran’s Q test. Significant outcomes from the Q test (*p* < 0.05) denote heterogeneity among IVs (24), with the results being visually presented through funnel plots. Pleiotropy was investigated through MR-Egger regression, which estimated the intercept term, referred to as the pleiotropic intercept, encapsulating the average pleiotropic impact of all genetic variations. A *p* value for the intercept exceeding 0.05 disregards the presence of pleiotropy (25).

### Statistical analysis

Statistical analysis was conducted using “TwoSampleMR,” “MR-PRESSO” and “forestplot” (26) in R version 4.3.1. Results were presented as odds ratios (ORs) along with their corresponding 95% confidence intervals (CIs). A significance level of *p* < 0.05 denoted a statistically significant difference between the two groups.

## Results

### Selection of IVs

Following meticulous screening, the number of SNPs utilized for each exposure and outcome risk varied from 9 to 23. The F statistics associated with the SNPs encompassed in the study all exceeded 10, affirming the absence of weak bias in the study results and thereby validating their reliability. The findings are illustrated in the Supplementary Tables S1–S3.

### Causal association of uIA with SCZ

**Figure 2:**
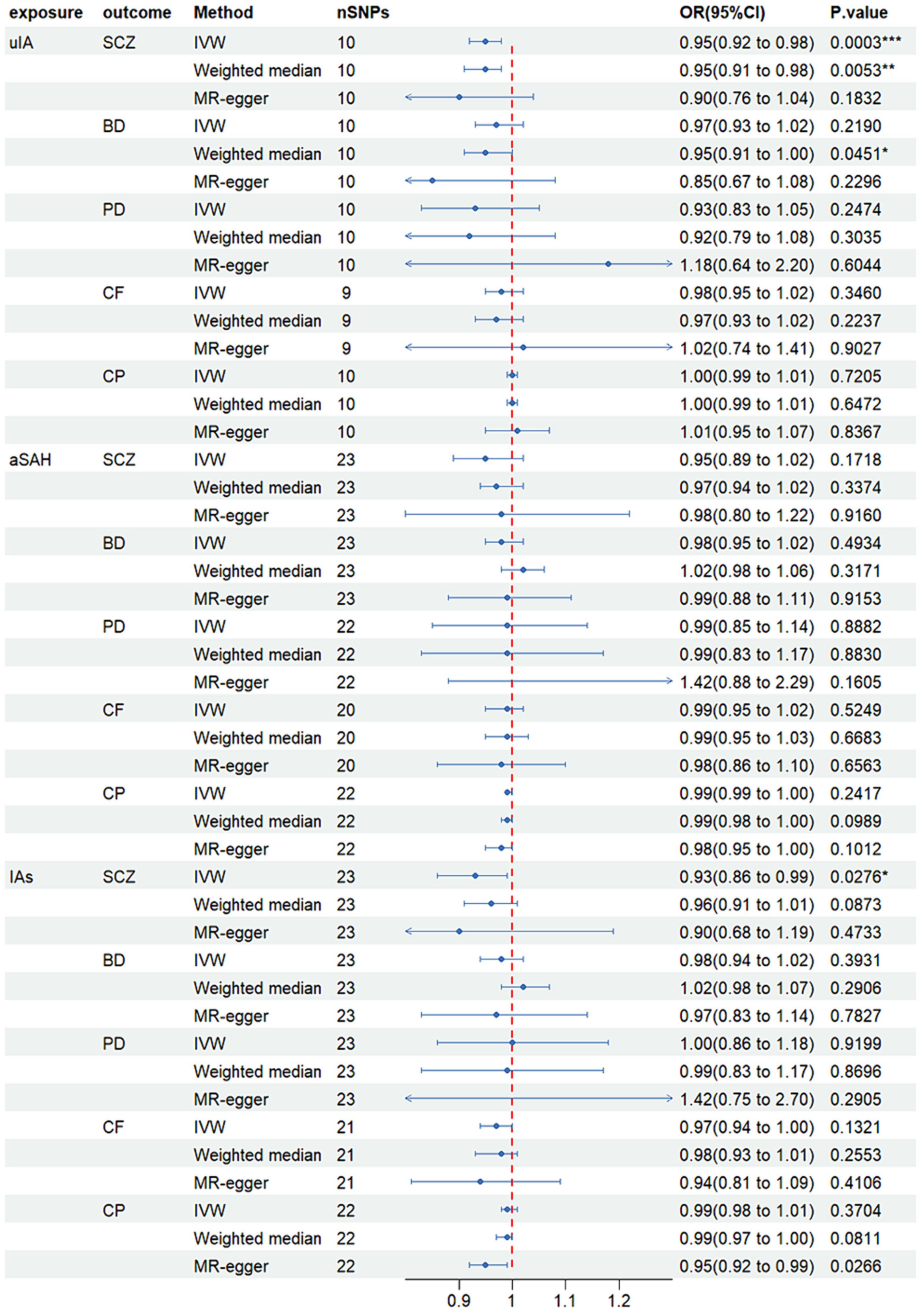
Mendelian randomized forest plot of causal effects between intracranial aneurysms, unruptured intracranial aneurysm and aneurysmal subarachnoid hemorrhage. *p < 0.05; **p < 0.01; ***p < 0.001.

MR analysis revealed that in the inverse-variance weighted (IVW) model, unruptured intracranial aneurysm (uIA) exhibited a causal association with schizophrenia (SCZ) (OR = 0.95, 95% CI 0.92–0.98, *p* = 0.0002), indicating a negative association between uIA and SCZ (Figure 2). The weighted median method yielded similar causal estimations (OR = 0.95, 95% CI 0.91–0.98, *p* = 0.005) (Figure 2). Despite providing consistent estimates, the MR-Egger methods did not attain statistical significance. To ensure the stability of the above findings, sensitivity analysis was conducted on the included single nucleotide polymorphisms (SNPs). Cochran’s Q test revealed no evidence of heterogeneity (*p* = 0.705), while the MR-Egger intercept (*p* = 0.448) indicated no significant pleiotropy (Table 2; Supplementary Figure S1A). Funnel plots displayed general symmetry across all SNPs, indicating that causal associations are less likely to be affected by potential bias (Supplementary Figure S1B). No SNPs of significance were identified to exert a notable impact on the causal association estimate following exclusion via the leave-one-out method (Supplementary Figure S1C).

**Table 2:** Heterogeneity and pleiotropy tests for the associations between IA and psychiatric disorders.

Exposure	Outcome	SNP (*n*)	Heterogeneity test		Pleiotropy test		
			Q	Q-pval	MR-Egger intercept	Se	*p*
uIA	SCZ	10	6.345	0.705	0.014	0.018	0.448
	BD	10	17.267	0.045	0.030	0.028	0.305
	PD	10	3.127	0.959	−0.056	0.072	0.458
	CF	9	9.726	0.285	−0.008	0.036	0.826
	CP	10	9.899	0.359	−0.001	0.007	0.878
aSAH	SCZ	23	149.857	<0.001	−0.007	0.019	0.729
	BD	23	36.688	0.026	−0.001	0.010	0.908
	PD	22	37.321	0.015	−0.065	0.042	0.130
	*CF*	20	25.938	0.132	0.003	0.010	0.785
	CP	20	23.127	0.337	0.003	0.002	0.176
IAs	SCZ	23	130.743	<0.001	0.004	0.022	0.849
	BD	23	35.382	0.035	0.001	0.013	0.944
	PD	23	37.100	0.023	−0.056	0.051	0.287
	*CF*	21	24.809	0.209	0.006	0.012	0.619
	CP	22	28.233	0.134	0.007	0.003	0.038

IAs, intracranial aneurysms; uIA, unruptured intracranial aneurysm; aSAH, aneurysmal subarachnoid hemorrhage; SCZ, schizophrenia; BD, bipolar disorder; PD, panic disorder; CF, cognitive function; CP, cognitive performance.

### Causal association of other models

Moreover, IAs were causally linked with SCZ (OR = 0.92, 95% CI 0.86–0.99, *p* = 0.028), with IAs exhibiting a negative association with SCZ (Figure 2). However, no significant disparity was observed between MR-Egger and weighted median methods. Although Cochran’s Q test indicated heterogeneity, the MR-Egger intercept (*p* = 0.849) demonstrated no significant pleiotropic effect (Table 2; Supplementary Figure S2).

Additionally, no noteworthy distinction was noted between other models (*p* > 0.05). Comprehensive results for other models, encompassing outcomes from the three causal estimation methods, pleiotropy tests, and heterogeneity assessments, are presented in Table 2 and Supplementary Figures S1–S15.

## Discussion

We used Mendelian randomization to systematically assess the causal relationship between genetic susceptibility to intracranial aneurysms and the risk of four psychiatric disorders. Gene prediction results indicated that uIA and IAs were linked to a reduced risk of SCZ, a finding further supported by sensitivity analysis. However, genetically predicted uIA, aSAH, and IAs showed no connection to the risk of BD, PD, and cognitive impairment (CI). These results improve the understanding of the etiological basis of these four psychiatric disorders.

Epidemiological studies have shown that the prevalence of unruptured aneurysms is uncertain, with approximately one in four individuals experiencing ruptures in their lifetime, leading to severe consequences (27). The potential susceptibility of individuals with intracranial aneurysms to psychiatric disorders has been a topic of ongoing debate. MR analysis has demonstrated a positive causal link between intracranial aneurysms and major depressive disorder (MDD) (9). However, our MR analysis aligned with previous studies and did not identify a positive causal association between patients with intracranial aneurysms and SCZ or BD (28). Interestingly, our findings suggest that IA acts as a protective factor against schizophrenia SCZ.

Regarding the negative causal relationship between IA and SCZ, we propose the following possibilities. Firstly, existing research has highlighted the significant involvement of the renin-angiotensin-aldosterone system (RAAS) in both SCZ pathogenesis and IA development. Specifically, a strong correlation has been established between angiotensin-converting enzyme (ACE) expression and SCZ (29). Continuous administration of angiotensin receptor blockers (ARBs) has shown effectiveness in reducing SCZ symptoms (30), a finding supported by animal model studies (31). The involvement of the RAAS in IA pathogenesis remains debated. Studies suggest that downregulation of local RAAS activity may influence vascular remodeling, potentially contributing to brain aneurysm formation and rupture (32). Conversely, research using mouse models of IA has demonstrated high expression of angiotensin II and angiotensin type 1 receptors within these aneurysms, with inhibition of local RAAS activity showing promise in preventing aneurysm rupture (33). Additionally, among hypertensive patients with IA, the use of RAAS inhibitors has been linked with a significant reduction in rupture risk (34). Consequently, further research is needed to determine whether RAAS plays a role in the protective effect of IA against SCZ. Moreover, studies have highlighted the correlation between SCZ severity and distinct patterns of cerebral hemodynamics (35, 36). Prefrontal blood volume decreases in SCZ but not in MDD, suggesting that a positive causal relationship between IA and MDD may be related to prefrontal blood volume changes (37). A systematic review and meta-analysis revealed that negative symptoms of SCZ are linked with cortical frontolimbic hypoperfusion, while positive symptoms are associated with hyperperfusion. Furthermore, male individuals with schizophrenia exhibit increased blood-oxygen-level-dependent (BOLD) activation in the cerebellum, temporal gyrus, and right precuneus cortex. Notably, there is an increased incidence of low-frequency fluctuation in cerebral blood flow within the frontal and parietal lobes, as well as the insular cortex among male patients, and in the hippocampus, parahippocampus, and lentiform nucleus among female patients (38). Hemodynamic alterations also play a crucial role in the initiation, progression, and rupture of IA (39). In patients with UIA, a significant decrease in the local gyrations index (LGI) has been observed in brain regions including the posterior cingulate gyrus, retrospenial cortex, cuneiform gyrus, and lingual gyrus of the right hemisphere (40), areas overlapping with those implicated in SCZ. Additionally, variations in local biosignatures of IA, leading to changes in the local microenvironment (41, 42), may serve as potential contributing factors, necessitating further investigations to validate this hypothesis.

Cognitive impairment is often considered a long-term outcome of aneurysmal subarachnoid hemorrhage (aSAH) (13). However, the effect of unruptured intracranial aneurysms (uIA) on cognitive function has been debated in previous studies. Some research suggests that treatment of uIA may impact cognitive function (43), with post-treatment cognitive impairment observed in uIA patients without a history of aSAH (44). On the other hand, other studies indicate that uIA treatment does not significantly affect overall neuropsychological function (45, 46). A prior study reported declines in word fluency, verbal recall, and executive function among individuals with both ruptured and unruptured aneurysms (47). Nevertheless, our study did not find direct evidence supporting a causal link between uIA, IAs, aSAH, and cognitive impairment (CI). Consequently, cognitive impairment following treatment in patients with intracranial aneurysms may result from indirect effects of other factors. Research has shown that cognitive and functional impairments in patients with aSAH could be related to diffuse brain injury and specific complications like vasospasm and increased intracranial pressure (48, 49). Moreover, the choice of various anesthetics might influence post-surgery cognitive function (50). The underlying mechanism could be associated with inflammation in the central nervous system following aSAH (51, 52). There is a general consensus that inflammation plays a role in the formation, progression, and rupture of intracranial aneurysms (53). A recent study reveals that CNS-associated macrophages are believed to significantly contribute to aneurysm progression, yet their influence on cognitive function in patients after aSAH requires further exploration (54). Therefore, we speculate that the impact of intracranial aneurysms on cognitive function may stem from multiple factors, including underlying disease, aneurysm location, anesthetic drugs, and surgical techniques, rather than being solely attributed to the aneurysm itself. Further investigation is necessary to validate these specific reasons.

This study presents several notable strengths. Firstly, it employs a two-sample MR analysis, using genotype randomization to determine the causal relationship between IA and select psychiatric disorders. Secondly, this design reduces potential biases from reverse causality and confounding factors present in conventional studies, thus facilitating causal inference. Thirdly, the F-statistic for each exposure exceeds 10, indicating the absence of weak instrument bias. Additionally, we conducted comprehensive sensitivity analyses to evaluate the robustness of the MR model hypotheses.

However, there are some limitations to consider. Firstly, our findings are derived solely from populations of European descent, thus caution must be exercised when generalizing these results to non-European populations, as contextual factors and ethnicity may influence outcomes. Secondly, larger and more comprehensive datasets may be necessary for studying intracranial aneurysms, as well as for addressing data limitations, such as those encountered in reverse Mendelian randomization analysis due to excessive SNP losses. While we employed methods to detect and adjust for pleiotropy, some unmeasured confounding may still influence our results. Additional sensitivity analyses, including methods to account for horizontal pleiotropy, would strengthen causal inference. In addition, Our MR analysis may not capture the full genetic architecture and multifactorial etiology of psychiatric disorders. Future research should consider polygenic risk scores and integrate multi-omic data to better understand the genetic underpinnings of these conditions. Finally, our analysis does not account for the dynamic progression of psychiatric disorders. Longitudinal studies and repeated measures MR approaches are needed to explore how genetic predispositions influence disease trajectories over time. MR studies primarily focus on genetic variants and do not account for environmental and epigenetic factors that play significant roles in psychiatric disorders. Future studies should integrate environmental exposures and epigenetic modifications to provide a more comprehensive understanding of disease etiology.

## Conclusion

In conclusion, our findings suggest that both uIA and IAs might serve as protective factors against SCZ. However, comprehensive validation studies are warranted to substantiate these findings and elucidate the underlying molecular mechanisms comprehensively.

## Data availability statement

The datasets presented in this study can be found in online repositories. The names of the repository/repositories and accession number(s) can be found in the article/Supplementary material.

## Ethics statement

Ethical review and approval was not required for the study on human participants in accordance with the local legislation and institutional requirements. Written informed consent from the patients/participants or patients/participants' legal guardian/next of kin was not required to participate in this study in accordance with the national legislation and the institutional requirements.

## Author contributions

SB: Conceptualization, Writing – original draft, Writing – review & editing, Data curation, Formal analysis, Investigation, Methodology, Software. ZX: Data curation, Formal analysis, Investigation, Writing – review & editing, Validation. SH: Formal analysis, Writing – review & editing, Methodology, Software. XH: Software, Writing – review & editing, Investigation. JY: Writing – review & editing, Conceptualization, Funding acquisition, Supervision, Writing – original draft. BZ: Conceptualization, Supervision, Writing – original draft, Writing – review & editing.

## Footnotes

^1^ https://figshare.com/articles/dataset/Intracranial_aneurysm_genome-wide_association_study_summary_statistics_2020/11303372

^2^ https://pgc.unc.edu/

^3^ https://gwas.mrcieu.ac.uk/

## Supplementary material

SUPPLEMENTARY FIGURE S1 Supplementary figures for uIA on SCZ. uIA, unruptured intracranial aneurysm; SCZ, schizophrenia; MR, Mendelian randomization; IV, instrumental variable; SE, standard error. **(A)** Scatter plot for uIA on SCZ; **(B)** funnel plot for uIA on SCZ; **(C)** leave-one-out graph for uIA on SCZ; **(D)** forest plot for uIA on SCZ.

SUPPLEMENTARY FIGURE S2 Supplementary figures for uIA on BD. uIA, unruptured intracranial aneurysm; BD, bipolar disorder; MR, Mendelian randomization; IV, instrumental variable; SE, standard error. **(A)** Scatter plot for uIA on BD; **(B)** funnel plot for uIA on BD; **(C)** leave-one-out graph for uIA on BD; **(D)** forest plot for uIA on BD.

SUPPLEMENTARY FIGURE S3 Supplementary figures for uIA on PD. uIA, unruptured intracranial aneurysm; PD, panic disorder; MR, Mendelian randomization; IV, instrumental variable; SE, standard error. **(A)** Scatter plot for uIA on PD; **(B)** funnel plot for uIA on PD; **(C)** leave-one-out graph for uIA on PD; **(D)** forest plot for uIA on PD.

SUPPLEMENTARY FIGURE S4 Supplementary figures for uIA on CF. uIA, unruptured intracranial aneurysm; CF, cognitive function; MR, Mendelian randomization; IV, instrumental variable; SE, standard error. **(A)** Scatter plot for uIA on CF; **(B)** Funnel plot for uIA on CF; **(C)** Leave-one-out graph for uIA on CF; **(D)** Forest plot for uIA on CF.

SUPPLEMENTARY FIGURE S5 Supplementary figures for uIA on CP. uIA, unruptured intracranial aneurysm; CP, cognitive performance; MR, Mendelian randomization; IV, instrumental variable; SE, standard error. **(A)** Scatter plot for uIA on CP; **(B)** funnel plot for uIA on CP; **(C)** leave-one-out graph for uIA on CP; **(D)** forest plot for uIA on CP.

SUPPLEMENTARY FIGURE S6 Supplementary figures for aSAH on SCZ. aSAH, aneurysmal subarachnoid hemorrhage; SCZ, schizophrenia; MR, Mendelian randomization; IV, instrumental variable; SE, standard error. **(A)** Scatter plot for SAH on SCZ; **(B)** funnel plot for SAH on SCZ; **(C)** leave-one-out graph for SAH on SCZ; **(D)** forest plot for SAH on SCZ.

SUPPLEMENTARY FIGURE S7 Supplementary figures for aSAH on BD. aSAH, aneurysmal subarachnoid hemorrhage; BD, bipolar disorder; MR, Mendelian randomization; IV, instrumental variable; SE, standard error. **(A)** Scatter plot for SAH on BD; **(B)** funnel plot for uIA on BD; **(C)** leave-one-out graph for SAH on BD; **(D)** forest plot for SAH on BD.

SUPPLEMENTARY FIGURE S8 Supplementary figures for aSAH on PD. aSAH, aneurysmal subarachnoid hemorrhage; PD, panic disorder; MR, Mendelian randomization; IV, instrumental variable; SE, standard error. **(A)** Scatter plot for SAH on PD; **(B)** funnel plot for SAH on PD; **(C)** leave-one-out graph for SAH on PD; **(D)** forest plot for SAH on PD.

SUPPLEMENTARY FIGURE S9 Supplementary figures for aSAH on CF. aSAH, aneurysmal subarachnoid hemorrhage; CF, cognitive function; MR, Mendelian randomization; IV, instrumental variable; SE, standard error. **(A)** Scatter plot for SAH on CF; **(B)** funnel plot for SAH on CF; **(C)** leave-one-out graph for SAH on CF; **(D)** forest plot for SAH on CF.

SUPPLEMENTARY FIGURE S10 Supplementary figures for aSAH on CP. aSAH, aneurysmal subarachnoid hemorrhage; CP, cognitive performance; MR, Mendelian randomization; IV, instrumental variable; SE, standard error. **(A)** Scatter plot for SAH on CP; **(B)** funnel plot for SAH on CP; **(C)** leave-one-out graph for SAH on CP; **(D)** forest plot for SAH on CP.

SUPPLEMENTARY FIGURE S11 Supplementary figures for IAs on SCZ. IAs, intracranial aneurysms; SCZ, schizophrenia; MR, Mendelian randomization; IV, instrumental variable; SE, standard error. **(A)** Scatter plot for IAs on SCZ; **(B)** funnel plot for IAs on SCZ; **(C)** leave-one-out graph for IAs on SCZ; **(D)** forest plot for IAs on SCZ.

SUPPLEMENTARY FIGURE S12 Supplementary figures for IAs on BD. IAs, intracranial aneurysms; BD, bipolar disorder; MR, Mendelian randomization; IV, instrumental variable; SE, standard error. **(A)** Scatter plot for IAs on BD; **(B)** funnel plot for IAs on BD; **(C)** leave-one-out graph for IAs on BD; **(D)** forest plot for IAs on BD.

SUPPLEMENTARY FIGURE S13 Supplementary figures for IAs on PD. IAs, intracranial aneurysms; PD, panic disorder; MR, Mendelian randomization; IV, instrumental variable; SE, standard error. **(A)** Scatter plot for IAs on PD; **(B)** funnel plot for IAs on PD; **(C)** leave-one-out graph for IAs on PD; **(D)** forest plot for IAs on PD.

SUPPLEMENTARY FIGURE S14 Supplementary figures for IAs on CF. IAs, intracranial aneurysms; CF, cognitive function; MR, Mendelian randomization; IV, instrumental variable; SE, standard error. **(A)** Scatter plot for IAs on CF; **(B)** funnel plot for IAs on CF; **(C)** leave-one-out graph for IAs on CF; **(D)** forest plot for IAs on CF.

SUPPLEMENTARY FIGURE S15 Supplementary figures for IAs on CP. IAs, intracranial aneurysms; CP, cognitive performance; MR, Mendelian randomization; IV, instrumental variable; SE, standard error. **(A)** Scatter plot for IAs on CP; **(B)** funnel plot for IAs on CP; **(C)** leave-one-out graph for IAs on CP; **(D)** forest plot for IAs on CP.
