# Supplementary material for: Association between psychiatric disorders and intracranial aneurysms: evidence from Mendelian randomization analysis
Source: Front Neurol. 2024 Jul 26;15:1422984. doi: 10.3389/fneur.2024.1422984 (PMC11312739; doi:10.3389/fneur.2024.1422984)
Supplement: Supplementary file 1 [file Table_1.docx]

**Supplementary Table S1.** Single SNP analysis for the causal association between uIA and psychiatric disorders

| **exposure** | **Outcome** | **SNP** | **Effect allele** | **Other allele** | **Beta** | **Se** | **P-value** | **F** |
| --- | --- | --- | --- | --- | --- | --- | --- | --- |
| uIA | Schizophrenia | rs10893077 | A | G | -0.2538 | 0.054 | 2.63E-06 | 22.09 |
| uIA | Schizophrenia | rs11646044 | T | G | -0.2051 | 0.040 | 2.53E-07 | 26.56 |
| uIA | Schizophrenia | rs11720244 | T | C | -0.2227 | 0.046 | 9.68E-07 | 23.96 |
| uIA | Schizophrenia | rs1537373 | T | G | -0.1954 | 0.034 | 1.08E-08 | 32.64 |
| uIA | Schizophrenia | rs2417658 | T | C | -0.2178 | 0.046 | 2.23E-06 | 22.42 |
| uIA | Schizophrenia | rs571138 | A | C | -0.2042 | 0.040 | 2.75E-07 | 26.46 |
| uIA | Schizophrenia | rs62349022 | T | C | -0.2468 | 0.052 | 1.84E-06 | 22.79 |
| uIA | Schizophrenia | rs6798962 | T | C | -0.1876 | 0.041 | 4.61E-06 | 21.04 |
| uIA | Schizophrenia | rs72705377 | A | G | -0.5121 | 0.109 | 2.86E-06 | 21.91 |
| uIA | Schizophrenia | rs77028772 | A | G | -0.2715 | 0.057 | 1.70E-06 | 22.93 |
| uIA | Bipolar Disorder | rs10893077 | A | G | -0.2538 | 0.054 | 2.63E-06 | 22.09 |
| uIA | Bipolar Disorder | rs11646044 | T | G | -0.2051 | 0.040 | 2.53E-07 | 26.56 |
| uIA | Bipolar Disorder | rs11720244 | T | C | -0.2227 | 0.046 | 9.68E-07 | 23.96 |
| uIA | Bipolar Disorder | rs1537373 | T | G | -0.1954 | 0.034 | 1.08E-08 | 32.64 |
| uIA | Bipolar Disorder | rs2417658 | T | C | -0.2178 | 0.046 | 2.23E-06 | 22.42 |
| uIA | Bipolar Disorder | rs571138 | A | C | -0.2042 | 0.040 | 2.75E-07 | 26.46 |
| uIA | Bipolar Disorder | rs62349022 | T | C | -0.2468 | 0.052 | 1.84E-06 | 22.79 |
| uIA | Bipolar Disorder | rs6798962 | T | C | -0.1876 | 0.041 | 4.61E-06 | 21.04 |
| uIA | Bipolar Disorder | rs72705377 | A | G | -0.5121 | 0.109 | 2.86E-06 | 21.91 |
| uIA | Bipolar Disorder | rs77028772 | A | G | -0.2715 | 0.057 | 1.70E-06 | 22.93 |
| uIA | Panic Disorder | rs10893077 | A | G | -0.2538 | 0.054 | 2.63E-06 | 22.09 |
| uIA | Panic Disorder | rs11646044 | T | G | -0.2051 | 0.040 | 2.53E-07 | 26.56 |
| uIA | Panic Disorder | rs11720244 | T | C | -0.2227 | 0.046 | 9.68E-07 | 23.96 |
| uIA | Panic Disorder | rs1537373 | T | G | -0.1954 | 0.034 | 1.08E-08 | 32.64 |
| uIA | Panic Disorder | rs2417658 | T | C | -0.2178 | 0.046 | 2.23E-06 | 22.42 |
| uIA | Panic Disorder | rs571138 | A | C | -0.2042 | 0.040 | 2.75E-07 | 26.46 |
| uIA | Panic Disorder | rs62349022 | T | C | -0.2468 | 0.052 | 1.84E-06 | 22.79 |
| uIA | Panic Disorder | rs6798962 | T | C | -0.1876 | 0.041 | 4.61E-06 | 21.04 |
| uIA | Panic Disorder | rs72705377 | A | G | -0.5121 | 0.109 | 2.86E-06 | 21.91 |
| uIA | Panic Disorder | rs77028772 | A | G | -0.2715 | 0.057 | 1.70E-06 | 22.93 |
| uIA | Cognitive function | rs10893077 | A | G | -0.2538 | 0.054 | 2.63E-06 | 22.09 |
| uIA | Cognitive function | rs11646044 | T | G | -0.2051 | 0.040 | 2.53E-07 | 26.56 |
| uIA | Cognitive function | rs11720244 | T | C | -0.2227 | 0.046 | 9.68E-07 | 23.96 |
| uIA | Cognitive function | rs1537373 | T | G | -0.1954 | 0.034 | 1.08E-08 | 32.64 |
| uIA | Cognitive function | rs2417658 | T | C | -0.2178 | 0.046 | 2.23E-06 | 22.42 |
| uIA | Cognitive function | rs571138 | A | C | -0.2042 | 0.040 | 2.75E-07 | 26.46 |
| uIA | Cognitive function | rs62349022 | T | C | -0.2468 | 0.052 | 1.84E-06 | 22.79 |
| uIA | Cognitive function | rs6798962 | T | C | -0.1876 | 0.041 | 4.61E-06 | 21.04 |
| uIA | Cognitive function | rs77028772 | A | G | -0.2715 | 0.057 | 1.70E-06 | 22.93 |
| uIA | Cognitive performance | rs10893077 | A | G | -0.2538 | 0.054 | 2.63E-06 | 22.09 |
| uIA | Cognitive performance | rs11646044 | T | G | -0.2051 | 0.040 | 2.53E-07 | 26.56 |
| uIA | Cognitive performance | rs11720244 | T | C | -0.2227 | 0.046 | 9.68E-07 | 23.96 |
| uIA | Cognitive performance | rs1537373 | T | G | -0.1954 | 0.034 | 1.08E-08 | 32.64 |
| uIA | Cognitive performance | rs2417658 | T | C | -0.2178 | 0.046 | 2.23E-06 | 22.42 |
| uIA | Cognitive performance | rs571138 | A | C | -0.2042 | 0.040 | 2.75E-07 | 26.46 |
| uIA | Cognitive performance | rs62349022 | T | C | -0.2468 | 0.052 | 1.84E-06 | 22.79 |
| uIA | Cognitive performance | rs6798962 | T | C | -0.1876 | 0.041 | 4.61E-06 | 21.04 |
| uIA | Cognitive performance | rs72705377 | A | G | -0.5121 | 0.109 | 2.86E-06 | 21.91 |
| uIA | Cognitive performance | rs77028772 | A | G | -0.2715 | 0.057 | 1.70E-06 | 22.93 |
