# Supplementary material for: Association between psychiatric disorders and intracranial aneurysms: evidence from Mendelian randomization analysis
Source: Front Neurol. 2024 Jul 26;15:1422984. doi: 10.3389/fneur.2024.1422984 (PMC11312739; doi:10.3389/fneur.2024.1422984)
Supplement: Supplementary file 2 [file Table_2.docx]

**Supplementary Table S2.** Single SNP analysis for the causal association between SAH and psychiatric disorders

| **exposure** | **Outcome** | **SNP** | **Effect allele** | **Other allele** | **Beta** | **Se** | **P-value** | **F** |
| --- | --- | --- | --- | --- | --- | --- | --- | --- |
| SAH | Schizophrenia | rs10519203 | A | G | -0.1187 | 0.024 | 4.88E-07 | 25.30 |
| SAH | Schizophrenia | rs10898038 | T | C | 0.1396 | 0.029 | 1.26E-06 | 23.50 |
| SAH | Schizophrenia | rs114624871 | T | C | 0.5156 | 0.110 | 2.96E-06 | 21.85 |
| SAH | Schizophrenia | rs11661542 | A | C | -0.1715 | 0.024 | 3.18E-13 | 53.26 |
| SAH | Schizophrenia | rs12125195 | T | C | 0.1976 | 0.037 | 7.54E-08 | 28.99 |
| SAH | Schizophrenia | rs12310399 | T | C | -0.1487 | 0.024 | 3.19E-10 | 39.70 |
| SAH | Schizophrenia | rs12924920 | A | G | -0.1273 | 0.027 | 2.04E-06 | 22.56 |
| SAH | Schizophrenia | rs13133009 | T | G | -0.3309 | 0.072 | 4.85E-06 | 20.89 |
| SAH | Schizophrenia | rs1537373 | T | G | -0.1908 | 0.023 | 4.84E-17 | 70.65 |
| SAH | Schizophrenia | rs28856948 | A | G | -0.1289 | 0.027 | 1.19E-06 | 23.66 |
| SAH | Schizophrenia | rs373971906 | T | G | 0.3699 | 0.077 | 1.45E-06 | 23.20 |
| SAH | Schizophrenia | rs39713 | T | C | 0.2011 | 0.040 | 3.50E-07 | 25.92 |
| SAH | Schizophrenia | rs4510361 | T | C | -0.1305 | 0.028 | 3.32E-06 | 21.57 |
| SAH | Schizophrenia | rs4705938 | T | C | 0.1086 | 0.023 | 1.38E-06 | 23.30 |
| SAH | Schizophrenia | rs62343027 | A | G | 0.3502 | 0.072 | 1.32E-06 | 23.40 |
| SAH | Schizophrenia | rs62516550 | T | C | 0.1783 | 0.027 | 2.94E-11 | 44.26 |
| SAH | Schizophrenia | rs6841581 | A | G | -0.2658 | 0.035 | 3.52E-14 | 57.35 |
| SAH | Schizophrenia | rs72841270 | T | G | 0.1768 | 0.035 | 3.98E-07 | 25.66 |
| SAH | Schizophrenia | rs75319757 | T | C | -0.2604 | 0.055 | 1.85E-06 | 22.75 |
| SAH | Schizophrenia | rs7543680 | A | G | 0.1348 | 0.028 | 1.09E-06 | 23.85 |
| SAH | Schizophrenia | rs79780963 | T | C | -0.2206 | 0.045 | 7.11E-07 | 24.57 |
| SAH | Schizophrenia | rs80242932 | T | C | -0.3263 | 0.071 | 4.24E-06 | 21.18 |
| SAH | Schizophrenia | rs9315204 | T | C | 0.1356 | 0.026 | 2.40E-07 | 26.58 |
| SAH | Bipolar Disorder | rs10519203 | A | G | -0.1187 | 0.024 | 4.88E-07 | 25.30 |
| SAH | Bipolar Disorder | rs10898038 | T | C | 0.1396 | 0.029 | 1.26E-06 | 23.50 |
| SAH | Bipolar Disorder | rs114624871 | T | C | 0.5156 | 0.110 | 2.96E-06 | 21.85 |
| SAH | Bipolar Disorder | rs11661542 | A | C | -0.1715 | 0.024 | 3.18E-13 | 53.26 |
| SAH | Bipolar Disorder | rs12125195 | T | C | 0.1976 | 0.037 | 7.54E-08 | 28.99 |
| SAH | Bipolar Disorder | rs12310399 | T | C | -0.1487 | 0.024 | 3.19E-10 | 39.70 |
| SAH | Bipolar Disorder | rs12924920 | A | G | -0.1273 | 0.027 | 2.04E-06 | 22.56 |
| SAH | Bipolar Disorder | rs13133009 | T | G | -0.3309 | 0.072 | 4.85E-06 | 20.89 |
| SAH | Bipolar Disorder | rs1537373 | T | G | -0.1908 | 0.023 | 4.84E-17 | 70.65 |
| SAH | Bipolar Disorder | rs28856948 | A | G | -0.1289 | 0.027 | 1.19E-06 | 23.66 |
| SAH | Bipolar Disorder | rs373971906 | T | G | 0.3699 | 0.077 | 1.45E-06 | 23.20 |
| SAH | Bipolar Disorder | rs39713 | T | C | 0.2011 | 0.040 | 3.50E-07 | 25.92 |
| SAH | Bipolar Disorder | rs4510361 | T | C | -0.1305 | 0.028 | 3.32E-06 | 21.57 |
| SAH | Bipolar Disorder | rs4705938 | T | C | 0.1086 | 0.023 | 1.38E-06 | 23.30 |
| SAH | Bipolar Disorder | rs62343027 | A | G | 0.3502 | 0.072 | 1.32E-06 | 23.40 |
| SAH | Bipolar Disorder | rs62516550 | T | C | 0.1783 | 0.027 | 2.94E-11 | 44.26 |
| SAH | Bipolar Disorder | rs6841581 | A | G | -0.2658 | 0.035 | 3.52E-14 | 57.35 |
| SAH | Bipolar Disorder | rs72841270 | T | G | 0.1768 | 0.035 | 3.98E-07 | 25.66 |
| SAH | Bipolar Disorder | rs75319757 | T | C | -0.2604 | 0.055 | 1.85E-06 | 22.75 |
| SAH | Bipolar Disorder | rs7543680 | A | G | 0.1348 | 0.028 | 1.09E-06 | 23.85 |
| SAH | Bipolar Disorder | rs79780963 | T | C | -0.2206 | 0.045 | 7.11E-07 | 24.57 |
| SAH | Bipolar Disorder | rs80242932 | T | C | -0.3263 | 0.071 | 4.24E-06 | 21.18 |
| SAH | Bipolar Disorder | rs9315204 | T | C | 0.1356 | 0.026 | 2.40E-07 | 26.58 |
| SAH | Panic Disorder | rs10519203 | A | G | -0.1187 | 0.024 | 4.88E-07 | 25.30 |
| SAH | Panic Disorder | rs10898038 | T | C | 0.1396 | 0.029 | 1.26E-06 | 23.50 |
| SAH | Panic Disorder | rs114624871 | T | C | 0.5156 | 0.110 | 2.96E-06 | 21.85 |
| SAH | Panic Disorder | rs11661542 | A | C | -0.1715 | 0.024 | 3.18E-13 | 53.26 |
| SAH | Panic Disorder | rs12125195 | T | C | 0.1976 | 0.037 | 7.54E-08 | 28.99 |
| SAH | Panic Disorder | rs12310399 | T | C | -0.1487 | 0.024 | 3.19E-10 | 39.70 |
| SAH | Panic Disorder | rs12924920 | A | G | -0.1273 | 0.027 | 2.04E-06 | 22.56 |
| SAH | Panic Disorder | rs13133009 | T | G | -0.3309 | 0.072 | 4.85E-06 | 20.89 |
| SAH | Panic Disorder | rs1537373 | T | G | -0.1908 | 0.023 | 4.84E-17 | 70.65 |
| SAH | Panic Disorder | rs28856948 | A | G | -0.1289 | 0.027 | 1.19E-06 | 23.66 |
| SAH | Panic Disorder | rs39713 | T | C | 0.2011 | 0.040 | 3.50E-07 | 25.92 |
| SAH | Panic Disorder | rs4510361 | T | C | -0.1305 | 0.028 | 3.32E-06 | 21.57 |
| SAH | Panic Disorder | rs4705938 | T | C | 0.1086 | 0.023 | 1.38E-06 | 23.30 |
| SAH | Panic Disorder | rs62343027 | A | G | 0.3502 | 0.072 | 1.32E-06 | 23.40 |
| SAH | Panic Disorder | rs62516550 | T | C | 0.1783 | 0.027 | 2.94E-11 | 44.26 |
| SAH | Panic Disorder | rs6841581 | A | G | -0.2658 | 0.035 | 3.52E-14 | 57.35 |
| SAH | Panic Disorder | rs72841270 | T | G | 0.1768 | 0.035 | 3.98E-07 | 25.66 |
| SAH | Panic Disorder | rs75319757 | T | C | -0.2604 | 0.055 | 1.85E-06 | 22.75 |
| SAH | Panic Disorder | rs7543680 | A | G | 0.1348 | 0.028 | 1.09E-06 | 23.85 |
| SAH | Panic Disorder | rs79780963 | T | C | -0.2206 | 0.045 | 7.11E-07 | 24.57 |
| SAH | Panic Disorder | rs80242932 | T | C | -0.3263 | 0.071 | 4.24E-06 | 21.18 |
| SAH | Panic Disorder | rs9315204 | T | C | 0.1356 | 0.026 | 2.40E-07 | 26.58 |
| SAH | Cognitive function | rs10519203 | A | G | -0.1187 | 0.024 | 4.88E-07 | 25.30 |
| SAH | Cognitive function | rs10898038 | T | C | 0.1396 | 0.029 | 1.26E-06 | 23.50 |
| SAH | Cognitive function | rs11661542 | A | C | -0.1715 | 0.024 | 3.18E-13 | 53.26 |
| SAH | Cognitive function | rs12125195 | T | C | 0.1976 | 0.037 | 7.54E-08 | 28.99 |
| SAH | Cognitive function | rs12310399 | T | C | -0.1487 | 0.024 | 3.19E-10 | 39.70 |
| SAH | Cognitive function | rs12924920 | A | G | -0.1273 | 0.027 | 2.04E-06 | 22.56 |
| SAH | Cognitive function | rs13133009 | T | G | -0.3309 | 0.072 | 4.85E-06 | 20.89 |
| SAH | Cognitive function | rs1537373 | T | G | -0.1908 | 0.023 | 4.84E-17 | 70.65 |
| SAH | Cognitive function | rs28856948 | A | G | -0.1289 | 0.027 | 1.19E-06 | 23.66 |
| SAH | Cognitive function | rs373971906 | T | G | 0.3699 | 0.077 | 1.45E-06 | 23.20 |
| SAH | Cognitive function | rs39713 | T | C | 0.2011 | 0.040 | 3.50E-07 | 25.92 |
| SAH | Cognitive function | rs4705938 | T | C | 0.1086 | 0.023 | 1.38E-06 | 23.30 |
| SAH | Cognitive function | rs62343027 | A | G | 0.3502 | 0.072 | 1.32E-06 | 23.40 |
| SAH | Cognitive function | rs62516550 | T | C | 0.1783 | 0.027 | 2.94E-11 | 44.26 |
| SAH | Cognitive function | rs6841581 | A | G | -0.2658 | 0.035 | 3.52E-14 | 57.35 |
| SAH | Cognitive function | rs75319757 | T | C | -0.2604 | 0.055 | 1.85E-06 | 22.75 |
| SAH | Cognitive function | rs7543680 | A | G | 0.1348 | 0.028 | 1.09E-06 | 23.85 |
| SAH | Cognitive function | rs79780963 | T | C | -0.2206 | 0.045 | 7.11E-07 | 24.57 |
| SAH | Cognitive function | rs80242932 | T | C | -0.3263 | 0.071 | 4.24E-06 | 21.18 |
| SAH | Cognitive function | rs9315204 | T | C | 0.1356 | 0.026 | 2.40E-07 | 26.58 |
| SAH | Cognitive performance | rs10519203 | A | G | -0.1187 | 0.024 | 4.88E-07 | 25.30 |
| SAH | Cognitive performance | rs10898038 | T | C | 0.1396 | 0.029 | 1.26E-06 | 23.50 |
| SAH | Cognitive performance | rs114624871 | T | C | 0.5156 | 0.110 | 2.96E-06 | 21.85 |
| SAH | Cognitive performance | rs11661542 | A | C | -0.1715 | 0.024 | 3.18E-13 | 53.26 |
| SAH | Cognitive performance | rs12125195 | T | C | 0.1976 | 0.037 | 7.54E-08 | 28.99 |
| SAH | Cognitive performance | rs12310399 | T | C | -0.1487 | 0.024 | 3.19E-10 | 39.70 |
| SAH | Cognitive performance | rs12924920 | A | G | -0.1273 | 0.027 | 2.04E-06 | 22.56 |
| SAH | Cognitive performance | rs13133009 | T | G | -0.3309 | 0.072 | 4.85E-06 | 20.89 |
| SAH | Cognitive performance | rs1537373 | T | G | -0.1908 | 0.023 | 4.84E-17 | 70.65 |
| SAH | Cognitive performance | rs28856948 | A | G | -0.1289 | 0.027 | 1.19E-06 | 23.66 |
| SAH | Cognitive performance | rs373971906 | T | G | 0.3699 | 0.077 | 1.45E-06 | 23.20 |
| SAH | Cognitive performance | rs39713 | T | C | 0.2011 | 0.040 | 3.50E-07 | 25.92 |
| SAH | Cognitive performance | rs4510361 | T | C | -0.1305 | 0.028 | 3.32E-06 | 21.57 |
| SAH | Cognitive performance | rs4705938 | T | C | 0.1086 | 0.023 | 1.38E-06 | 23.30 |
| SAH | Cognitive performance | rs62343027 | A | G | 0.3502 | 0.072 | 1.32E-06 | 23.40 |
| SAH | Cognitive performance | rs62516550 | T | C | 0.1783 | 0.027 | 2.94E-11 | 44.26 |
| SAH | Cognitive performance | rs6841581 | A | G | -0.2658 | 0.035 | 3.52E-14 | 57.35 |
| SAH | Cognitive performance | rs72841270 | T | G | 0.1768 | 0.035 | 3.98E-07 | 25.66 |
| SAH | Cognitive performance | rs75319757 | T | C | -0.2604 | 0.055 | 1.85E-06 | 22.75 |
| SAH | Cognitive performance | rs7543680 | A | G | 0.1348 | 0.028 | 1.09E-06 | 23.85 |
| SAH | Cognitive performance | rs80242932 | T | C | -0.3263 | 0.071 | 4.24E-06 | 21.18 |
| SAH | Cognitive performance | rs9315204 | T | C | 0.1356 | 0.026 | 2.40E-07 | 26.58 |
