# Supplementary material for: Association between psychiatric disorders and intracranial aneurysms: evidence from Mendelian randomization analysis
Source: Front Neurol. 2024 Jul 26;15:1422984. doi: 10.3389/fneur.2024.1422984 (PMC11312739; doi:10.3389/fneur.2024.1422984)
Supplement: Supplementary file 3 [file Table_3.docx]

**Supplementary Table S3.** Single SNP analysis for the causal association between IAs and psychiatric disorders

| **exposure** | **Outcome** | **SNP** | **Effect allele** | **Other allele** | **Beta** | **Se** | **P-value** | **F** |
| --- | --- | --- | --- | --- | --- | --- | --- | --- |
| IAs | Schizophrenia | rs10109988 | T | G | -0.1152 | 0.024 | 2.41E-06 | 22.29 |
| IAs | Schizophrenia | rs10519203 | A | G | -0.1203 | 0.020 | 1.29E-09 | 36.91 |
| IAs | Schizophrenia | rs111834968 | A | G | -0.1659 | 0.036 | 3.43E-06 | 21.60 |
| IAs | Schizophrenia | rs11646044 | T | G | -0.149 | 0.023 | 5.21E-11 | 43.08 |
| IAs | Schizophrenia | rs11661542 | A | C | -0.1659 | 0.021 | 5.74E-16 | 65.49 |
| IAs | Schizophrenia | rs12310399 | T | C | -0.1383 | 0.020 | 3.25E-12 | 48.79 |
| IAs | Schizophrenia | rs12925894 | A | G | -0.1279 | 0.028 | 3.47E-06 | 21.47 |
| IAs | Schizophrenia | rs1537373 | T | G | -0.1864 | 0.019 | 2.60E-22 | 94.25 |
| IAs | Schizophrenia | rs2616407 | T | C | -0.1461 | 0.027 | 5.91E-08 | 29.28 |
| IAs | Schizophrenia | rs28856948 | A | G | -0.1082 | 0.022 | 7.76E-07 | 24.41 |
| IAs | Schizophrenia | rs2983896 | A | G | 0.153 | 0.029 | 1.80E-07 | 27.27 |
| IAs | Schizophrenia | rs3742321 | T | C | -0.1475 | 0.022 | 4.10E-11 | 43.75 |
| IAs | Schizophrenia | rs39713 | T | C | 0.1823 | 0.033 | 4.10E-08 | 30.15 |
| IAs | Schizophrenia | rs4705938 | T | C | 0.1198 | 0.019 | 2.55E-10 | 40.18 |
| IAs | Schizophrenia | rs55965782 | T | C | 0.2644 | 0.031 | 9.03E-18 | 73.69 |
| IAs | Schizophrenia | rs56276323 | T | C | -0.3062 | 0.064 | 1.67E-06 | 22.96 |
| IAs | Schizophrenia | rs6997005 | A | G | 0.1489 | 0.019 | 1.26E-14 | 59.52 |
| IAs | Schizophrenia | rs72841270 | T | G | 0.1735 | 0.030 | 1.11E-08 | 32.57 |
| IAs | Schizophrenia | rs7668383 | T | C | -0.1347 | 0.027 | 6.39E-07 | 24.71 |
| IAs | Schizophrenia | rs7867949 | T | G | 0.2534 | 0.047 | 5.19E-08 | 29.70 |
| IAs | Schizophrenia | rs7934320 | A | G | -0.3937 | 0.080 | 9.65E-07 | 23.98 |
| IAs | Schizophrenia | rs79780963 | T | C | -0.2254 | 0.039 | 6.82E-09 | 33.57 |
| IAs | Schizophrenia | rs9977093 | A | G | 0.1407 | 0.030 | 3.18E-06 | 21.71 |
| IAs | Bipolar Disorder | rs10109988 | T | G | -0.1152 | 0.024 | 2.41E-06 | 22.29 |
| IAs | Bipolar Disorder | rs10519203 | A | G | -0.1203 | 0.020 | 1.29E-09 | 36.91 |
| IAs | Bipolar Disorder | rs111834968 | A | G | -0.1659 | 0.036 | 3.43E-06 | 21.60 |
| IAs | Bipolar Disorder | rs11646044 | T | G | -0.149 | 0.023 | 5.21E-11 | 43.08 |
| IAs | Bipolar Disorder | rs11661542 | A | C | -0.1659 | 0.021 | 5.74E-16 | 65.49 |
| IAs | Bipolar Disorder | rs12310399 | T | C | -0.1383 | 0.020 | 3.25E-12 | 48.79 |
| IAs | Bipolar Disorder | rs12925894 | A | G | -0.1279 | 0.028 | 3.47E-06 | 21.47 |
| IAs | Bipolar Disorder | rs1537373 | T | G | -0.1864 | 0.019 | 2.60E-22 | 94.25 |
| IAs | Bipolar Disorder | rs2616407 | T | C | -0.1461 | 0.027 | 5.91E-08 | 29.28 |
| IAs | Bipolar Disorder | rs28856948 | A | G | -0.1082 | 0.022 | 7.76E-07 | 24.41 |
| IAs | Bipolar Disorder | rs2983896 | A | G | 0.153 | 0.029 | 1.80E-07 | 27.27 |
| IAs | Bipolar Disorder | rs3742321 | T | C | -0.1475 | 0.022 | 4.10E-11 | 43.75 |
| IAs | Bipolar Disorder | rs39713 | T | C | 0.1823 | 0.033 | 4.10E-08 | 30.15 |
| IAs | Bipolar Disorder | rs4705938 | T | C | 0.1198 | 0.019 | 2.55E-10 | 40.18 |
| IAs | Bipolar Disorder | rs55965782 | T | C | 0.2644 | 0.031 | 9.03E-18 | 73.69 |
| IAs | Bipolar Disorder | rs56276323 | T | C | -0.3062 | 0.064 | 1.67E-06 | 22.96 |
| IAs | Bipolar Disorder | rs6997005 | A | G | 0.1489 | 0.019 | 1.26E-14 | 59.52 |
| IAs | Bipolar Disorder | rs72841270 | T | G | 0.1735 | 0.030 | 1.11E-08 | 32.57 |
| IAs | Bipolar Disorder | rs7668383 | T | C | -0.1347 | 0.027 | 6.39E-07 | 24.71 |
| IAs | Bipolar Disorder | rs7867949 | T | G | 0.2534 | 0.047 | 5.19E-08 | 29.70 |
| IAs | Bipolar Disorder | rs7934320 | A | G | -0.3937 | 0.080 | 9.65E-07 | 23.98 |
| IAs | Bipolar Disorder | rs79780963 | T | C | -0.2254 | 0.039 | 6.82E-09 | 33.57 |
| IAs | Bipolar Disorder | rs9977093 | A | G | 0.1407 | 0.030 | 3.18E-06 | 21.71 |
| IAs | Panic Disorder | rs10519203 | A | G | -0.1187 | 0.024 | 4.88E-07 | 25.30 |
| IAs | Panic Disorder | rs10109988 | T | G | -0.1152 | 0.024 | 2.41E-06 | 22.29 |
| IAs | Panic Disorder | rs10519203 | A | G | -0.1203 | 0.020 | 1.29E-09 | 36.91 |
| IAs | Panic Disorder | rs111834968 | A | G | -0.1659 | 0.036 | 3.43E-06 | 21.60 |
| IAs | Panic Disorder | rs11646044 | T | G | -0.149 | 0.023 | 5.21E-11 | 43.08 |
| IAs | Panic Disorder | rs11661542 | A | C | -0.1659 | 0.021 | 5.74E-16 | 65.49 |
| IAs | Panic Disorder | rs12310399 | T | C | -0.1383 | 0.020 | 3.25E-12 | 48.79 |
| IAs | Panic Disorder | rs12925894 | A | G | -0.1279 | 0.028 | 3.47E-06 | 21.47 |
| IAs | Panic Disorder | rs1537373 | T | G | -0.1864 | 0.019 | 2.60E-22 | 94.25 |
| IAs | Panic Disorder | rs2616407 | T | C | -0.1461 | 0.027 | 5.91E-08 | 29.28 |
| IAs | Panic Disorder | rs28856948 | A | G | -0.1082 | 0.022 | 7.76E-07 | 24.41 |
| IAs | Panic Disorder | rs2983896 | A | G | 0.153 | 0.029 | 1.80E-07 | 27.27 |
| IAs | Panic Disorder | rs3742321 | T | C | -0.1475 | 0.022 | 4.10E-11 | 43.75 |
| IAs | Panic Disorder | rs39713 | T | C | 0.1823 | 0.033 | 4.10E-08 | 30.15 |
| IAs | Panic Disorder | rs4705938 | T | C | 0.1198 | 0.019 | 2.55E-10 | 40.18 |
| IAs | Panic Disorder | rs55965782 | T | C | 0.2644 | 0.031 | 9.03E-18 | 73.69 |
| IAs | Panic Disorder | rs56276323 | T | C | -0.3062 | 0.064 | 1.67E-06 | 22.96 |
| IAs | Panic Disorder | rs6997005 | A | G | 0.1489 | 0.019 | 1.26E-14 | 59.52 |
| IAs | Panic Disorder | rs72841270 | T | G | 0.1735 | 0.030 | 1.11E-08 | 32.57 |
| IAs | Panic Disorder | rs7668383 | T | C | -0.1347 | 0.027 | 6.39E-07 | 24.71 |
| IAs | Panic Disorder | rs7867949 | T | G | 0.2534 | 0.047 | 5.19E-08 | 29.70 |
| IAs | Panic Disorder | rs7934320 | A | G | -0.3937 | 0.080 | 9.65E-07 | 23.98 |
| IAs | Cognitive function | rs79780963 | T | C | -0.2254 | 0.039 | 6.82E-09 | 33.57 |
| IAs | Cognitive function | rs9977093 | A | G | 0.1407 | 0.030 | 3.18E-06 | 21.71 |
| IAs | Cognitive function | rs10109988 | T | G | -0.1152 | 0.024 | 2.41E-06 | 22.29 |
| IAs | Cognitive function | rs10519203 | A | G | -0.1203 | 0.020 | 1.29E-09 | 36.91 |
| IAs | Cognitive function | rs111834968 | A | G | -0.1659 | 0.036 | 3.43E-06 | 21.60 |
| IAs | Cognitive function | rs11646044 | T | G | -0.149 | 0.023 | 5.21E-11 | 43.08 |
| IAs | Cognitive function | rs11661542 | A | C | -0.1659 | 0.021 | 5.74E-16 | 65.49 |
| IAs | Cognitive function | rs12310399 | T | C | -0.1383 | 0.020 | 3.25E-12 | 48.79 |
| IAs | Cognitive function | rs12925894 | A | G | -0.1279 | 0.028 | 3.47E-06 | 21.47 |
| IAs | Cognitive function | rs1537373 | T | G | -0.1864 | 0.019 | 2.60E-22 | 94.25 |
| IAs | Cognitive function | rs2616407 | T | C | -0.1461 | 0.027 | 5.91E-08 | 29.28 |
| IAs | Cognitive function | rs28856948 | A | G | -0.1082 | 0.022 | 7.76E-07 | 24.41 |
| IAs | Cognitive function | rs2983896 | A | G | 0.153 | 0.029 | 1.80E-07 | 27.27 |
| IAs | Cognitive function | rs3742321 | T | C | -0.1475 | 0.022 | 4.10E-11 | 43.75 |
| IAs | Cognitive function | rs39713 | T | C | 0.1823 | 0.033 | 4.10E-08 | 30.15 |
| IAs | Cognitive function | rs4705938 | T | C | 0.1198 | 0.019 | 2.55E-10 | 40.18 |
| IAs | Cognitive function | rs55965782 | T | C | 0.2644 | 0.031 | 9.03E-18 | 73.69 |
| IAs | Cognitive function | rs56276323 | T | C | -0.3062 | 0.064 | 1.67E-06 | 22.96 |
| IAs | Cognitive function | rs6997005 | A | G | 0.1489 | 0.019 | 1.26E-14 | 59.52 |
| IAs | Cognitive function | rs7668383 | T | C | -0.1347 | 0.027 | 6.39E-07 | 24.71 |
| IAs | Cognitive function | rs7867949 | T | G | 0.2534 | 0.047 | 5.19E-08 | 29.70 |
| IAs | Cognitive function | rs79780963 | T | C | -0.2254 | 0.039 | 6.82E-09 | 33.57 |
| IAs | Cognitive function | rs9977093 | A | G | 0.1407 | 0.030 | 3.18E-06 | 21.71 |
| IAs | Cognitive performance | rs10109988 | T | G | -0.1152 | 0.024 | 2.41E-06 | 22.29 |
| IAs | Cognitive performance | rs10519203 | A | G | -0.1203 | 0.020 | 1.29E-09 | 36.91 |
| IAs | Cognitive performance | rs111834968 | A | G | -0.1659 | 0.036 | 3.43E-06 | 21.60 |
| IAs | Cognitive performance | rs11646044 | T | G | -0.149 | 0.023 | 5.21E-11 | 43.08 |
| IAs | Cognitive performance | rs11661542 | A | C | -0.1659 | 0.021 | 5.74E-16 | 65.49 |
| IAs | Cognitive performance | rs12310399 | T | C | -0.1383 | 0.020 | 3.25E-12 | 48.79 |
| IAs | Cognitive performance | rs12925894 | A | G | -0.1279 | 0.028 | 3.47E-06 | 21.47 |
| IAs | Cognitive performance | rs1537373 | T | G | -0.1864 | 0.019 | 2.60E-22 | 94.25 |
| IAs | Cognitive performance | rs2616407 | T | C | -0.1461 | 0.027 | 5.91E-08 | 29.28 |
| IAs | Cognitive performance | rs28856948 | A | G | -0.1082 | 0.022 | 7.76E-07 | 24.41 |
| IAs | Cognitive performance | rs2983896 | A | G | 0.153 | 0.029 | 1.80E-07 | 27.27 |
| IAs | Cognitive performance | rs3742321 | T | C | -0.1475 | 0.022 | 4.10E-11 | 43.75 |
| IAs | Cognitive performance | rs39713 | T | C | 0.1823 | 0.033 | 4.10E-08 | 30.15 |
| IAs | Cognitive performance | rs4705938 | T | C | 0.1198 | 0.019 | 2.55E-10 | 40.18 |
| IAs | Cognitive performance | rs55965782 | T | C | 0.2644 | 0.031 | 9.03E-18 | 73.69 |
| IAs | Cognitive performance | rs56276323 | T | C | -0.3062 | 0.064 | 1.67E-06 | 22.96 |
| IAs | Cognitive performance | rs6997005 | A | G | 0.1489 | 0.019 | 1.26E-14 | 59.52 |
| IAs | Cognitive performance | rs72841270 | T | G | 0.1735 | 0.030 | 1.11E-08 | 32.57 |
| IAs | Cognitive performance | rs7668383 | T | C | -0.1347 | 0.027 | 6.39E-07 | 24.71 |
| IAs | Cognitive performance | rs7867949 | T | G | 0.2534 | 0.047 | 5.19E-08 | 29.70 |
| IAs | Cognitive performance | rs7934320 | A | G | -0.3937 | 0.080 | 9.65E-07 | 23.98 |
| IAs | Cognitive performance | rs9977093 | A | G | 0.1407 | 0.030 | 3.18E-06 | 21.71 |
