# Supplementary figures and images for: Association between psychiatric disorders and intracranial aneurysms: evidence from Mendelian randomization analysis
Source: Front Neurol. 2024 Jul 26;15:1422984. doi: 10.3389/fneur.2024.1422984 (PMC11312739; doi:10.3389/fneur.2024.1422984)

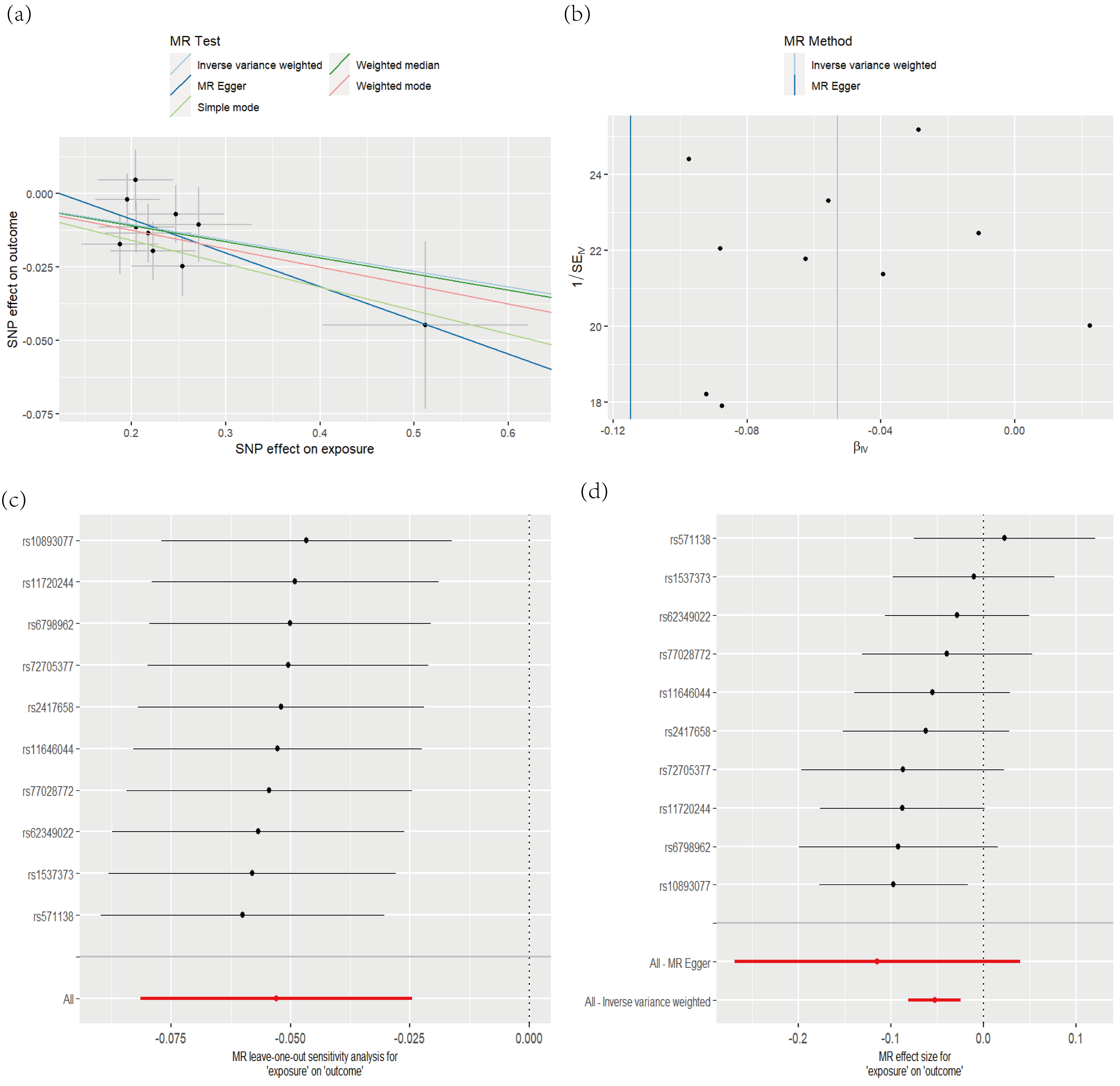

Supplement: SUPPLEMENTARY FIGURE S1 — Supplementary figures for uIA on SCZ. uIA, unruptured intracranial aneurysm; SCZ, schizophrenia; MR, Mendelian randomization; IV, instrumental variable; SE, standard error. (A) Scatter plot for uIA on SCZ; (B) funnel plot for uIA on SCZ; (C) leave-one-out graph for uIA on SCZ; (D) forest plot for uIA on SCZ. [file Image_1.TIF]

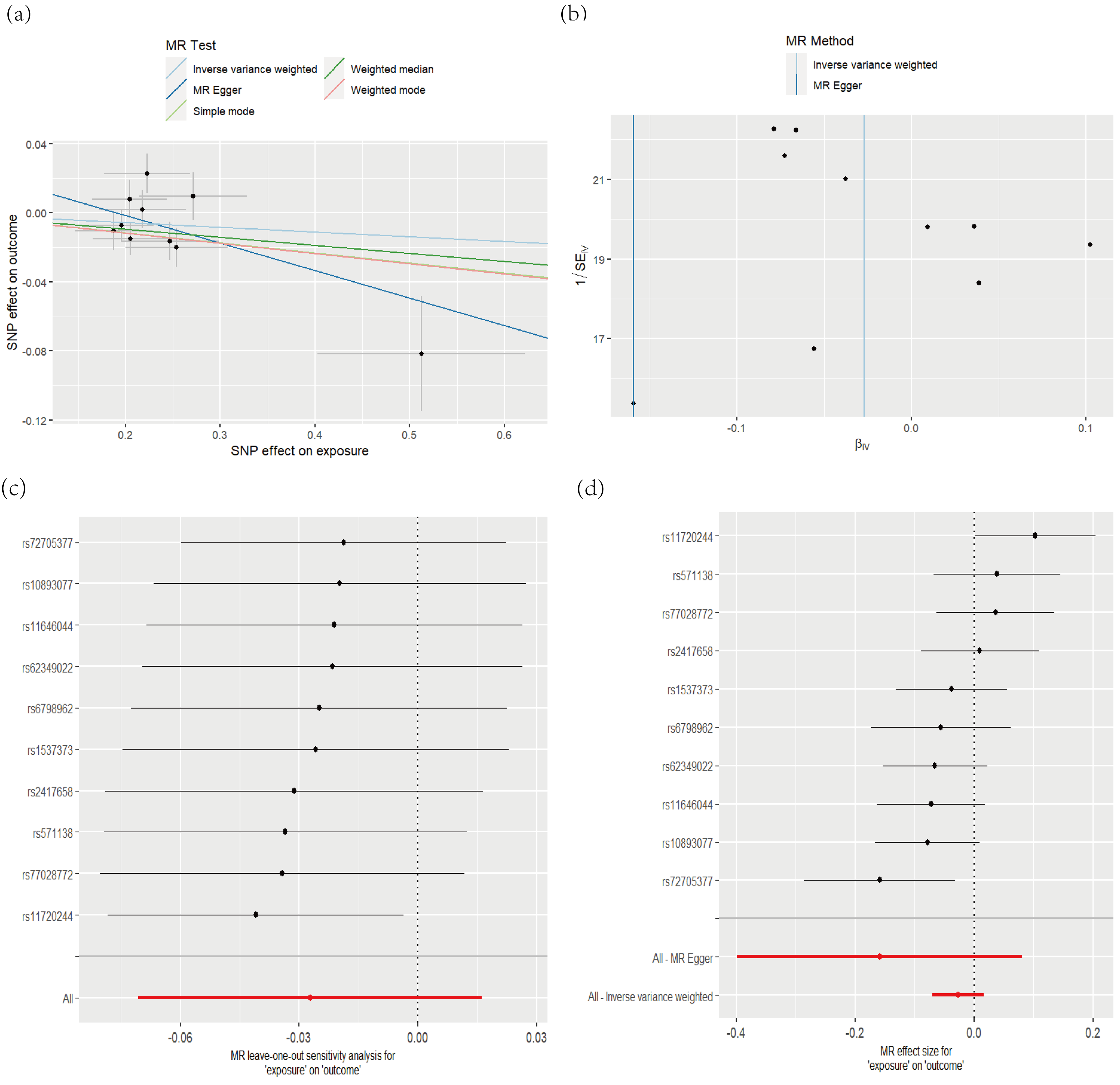

Supplement: SUPPLEMENTARY FIGURE S2 — Supplementary figures for uIA on BD. uIA, unruptured intracranial aneurysm; BD, bipolar disorder; MR, Mendelian randomization; IV, instrumental variable; SE, standard error. (A) Scatter plot for uIA on BD; (B) funnel plot for uIA on BD; (C) leave-one-out graph for uIA on BD; (D) forest plot for uIA on BD. [file Image_2.TIF]

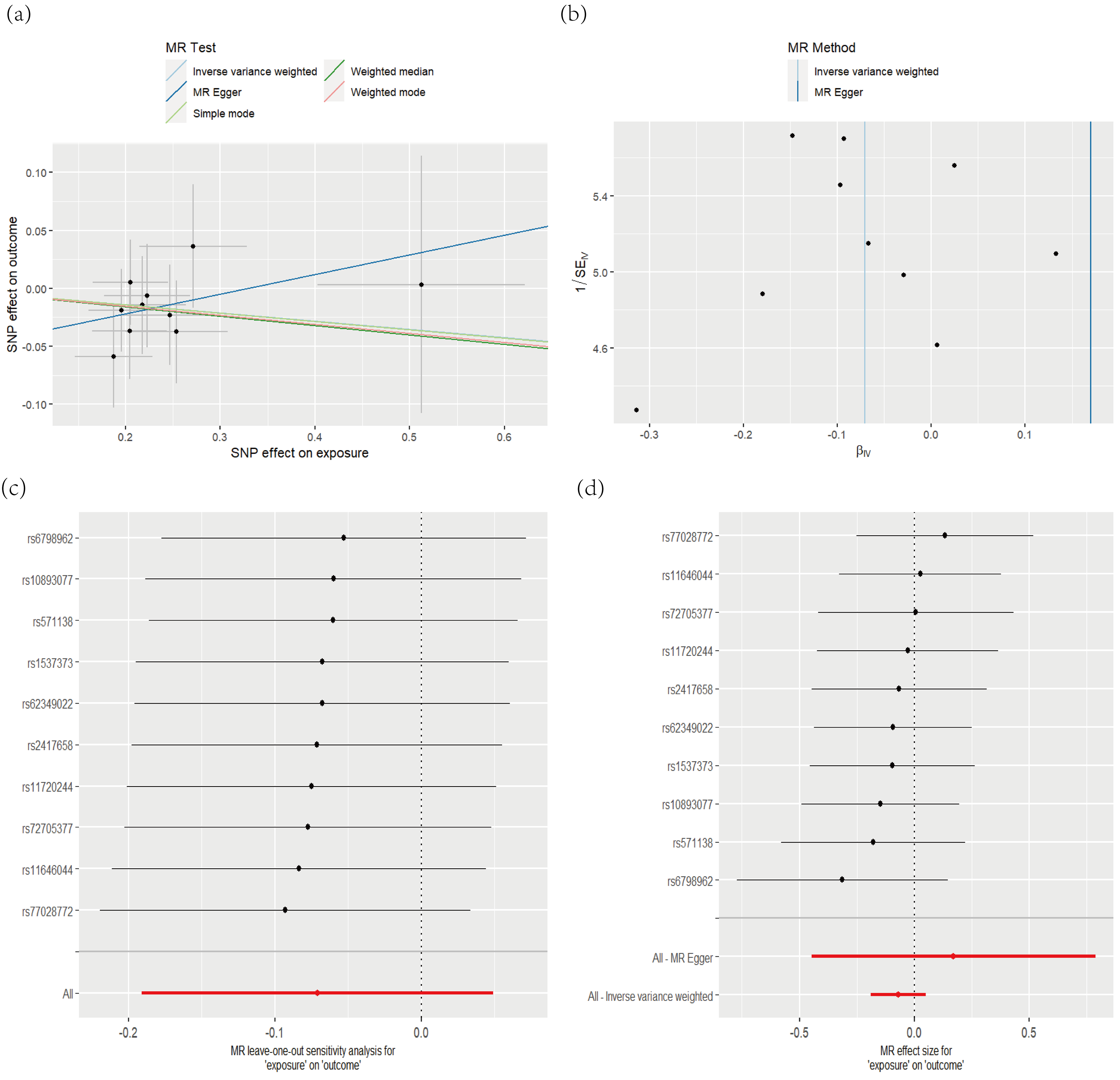

Supplement: SUPPLEMENTARY FIGURE S3 — Supplementary figures for uIA on PD. uIA, unruptured intracranial aneurysm; PD, panic disorder; MR, Mendelian randomization; IV, instrumental variable; SE, standard error. (A) Scatter plot for uIA on PD; (B) funnel plot for uIA on PD; (C) leave-one-out graph for uIA on PD; (D) forest plot for uIA on PD. [file Image_3.TIF]

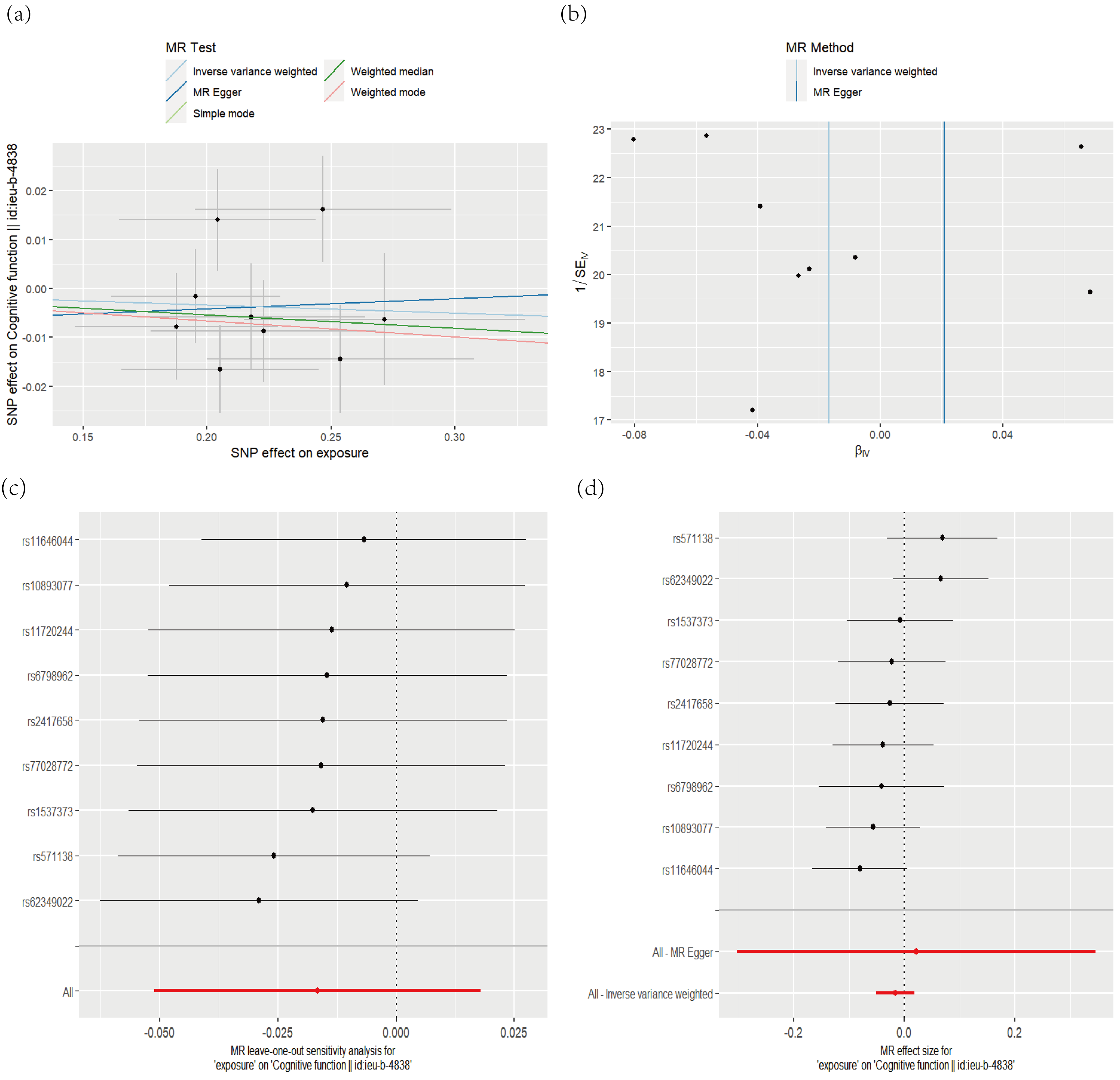

Supplement: SUPPLEMENTARY FIGURE S4 — Supplementary figures for uIA on CF. uIA, unruptured intracranial aneurysm; CF, cognitive function; MR, Mendelian randomization; IV, instrumental variable; SE, standard error. (A) Scatter plot for uIA on CF; (B) Funnel plot for uIA on CF; (C) Leave-one-out graph for uIA on CF; (D) Forest plot for uIA on CF. [file Image_4.TIF]

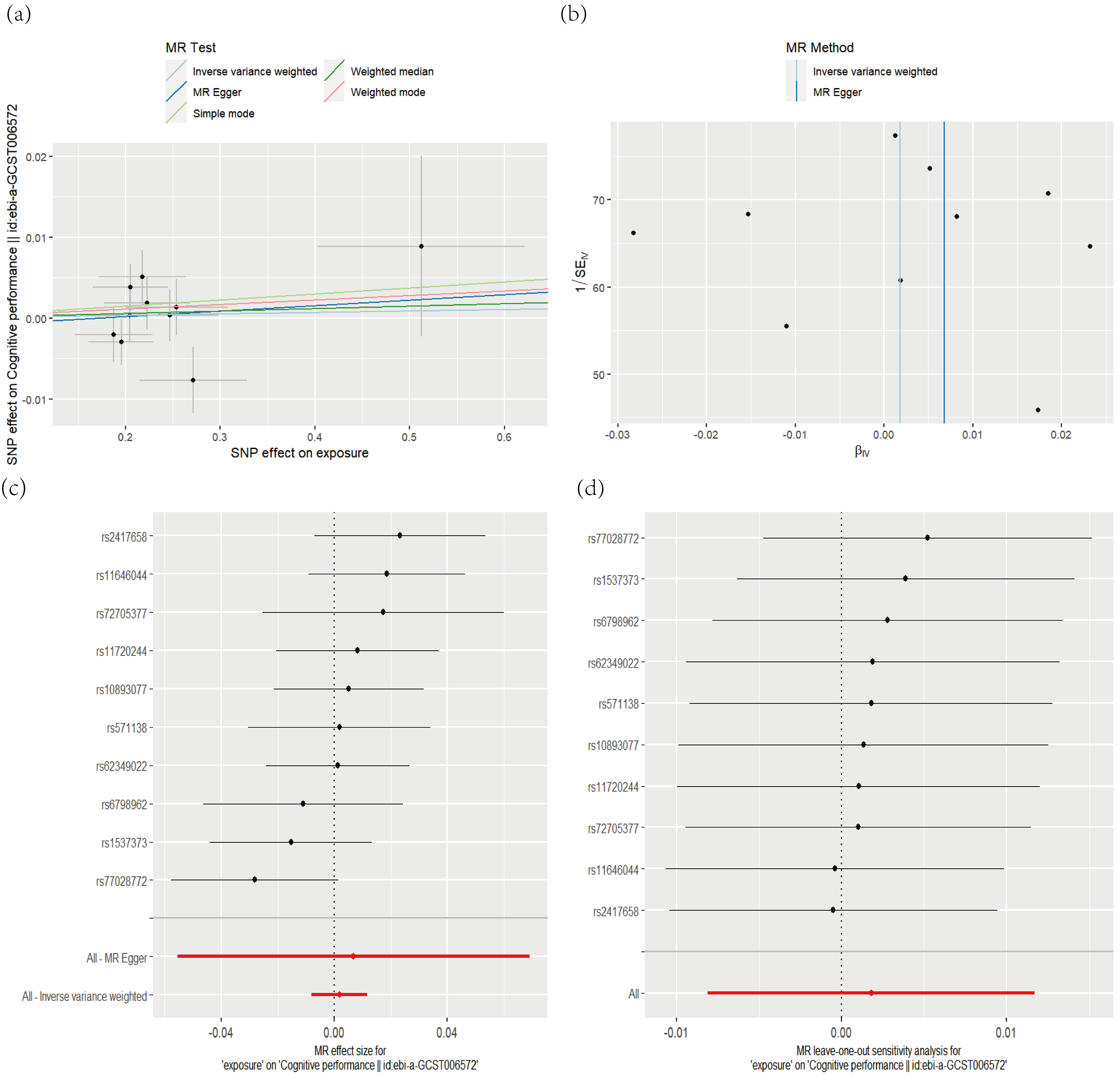

Supplement: SUPPLEMENTARY FIGURE S5 — Supplementary figures for uIA on CP. uIA, unruptured intracranial aneurysm; CP, cognitive performance; MR, Mendelian randomization; IV, instrumental variable; SE, standard error. (A) Scatter plot for uIA on CP; (B) funnel plot for uIA on CP; (C) leave-one-out graph for uIA on CP; (D) forest plot for uIA on CP. [file Image_5.TIF]

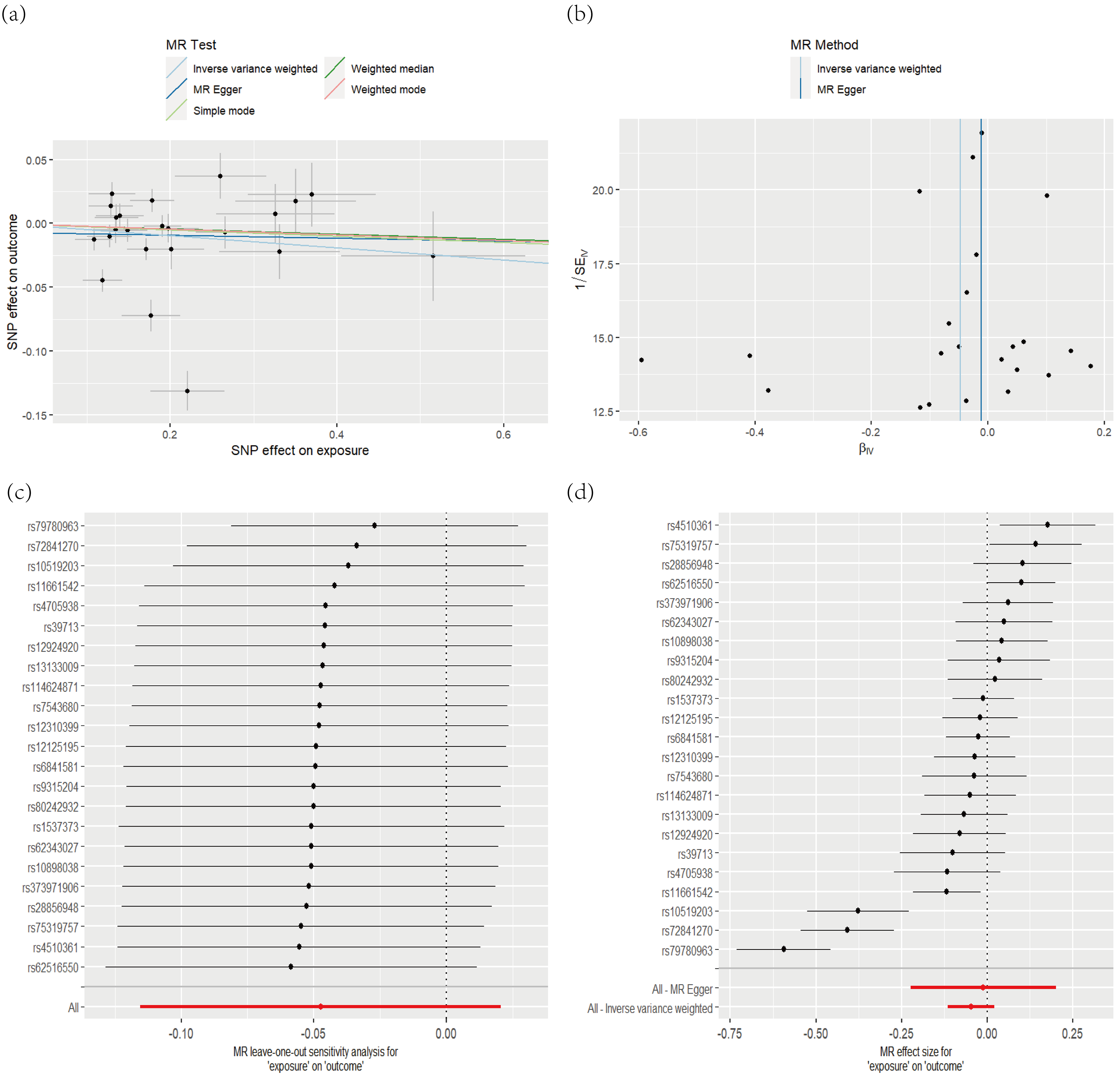

Supplement: SUPPLEMENTARY FIGURE S6 — Supplementary figures for aSAH on SCZ. aSAH, aneurysmal subarachnoid hemorrhage; SCZ, schizophrenia; MR, Mendelian randomization; IV, instrumental variable; SE, standard error. (A) Scatter plot for SAH on SCZ; (B) funnel plot for SAH on SCZ; (C) leave-one-out graph for SAH on SCZ; (D) forest plot for SAH on SCZ. [file Image_6.TIF]

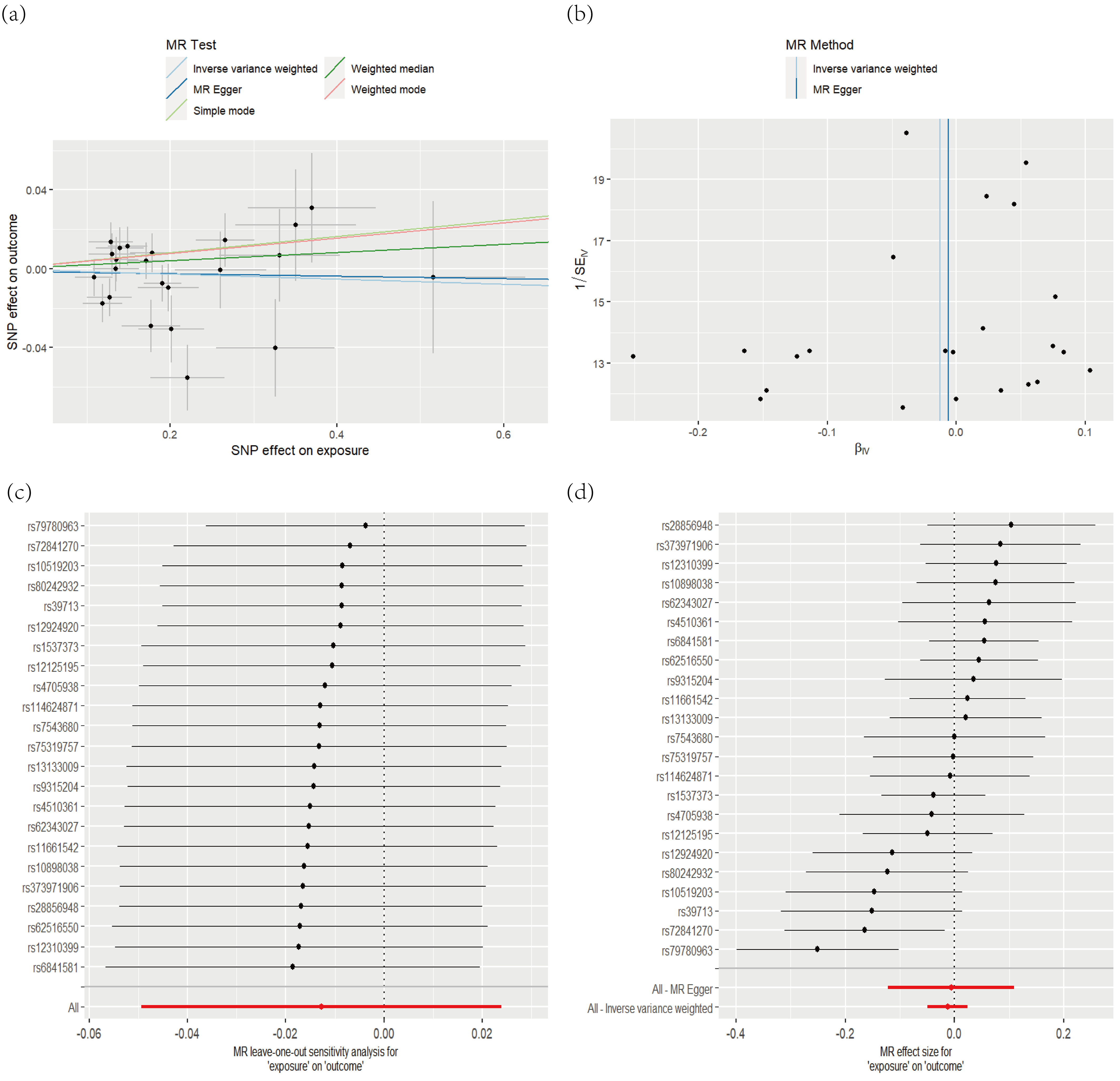

Supplement: SUPPLEMENTARY FIGURE S7 — Supplementary figures for aSAH on BD. aSAH, aneurysmal subarachnoid hemorrhage; BD, bipolar disorder; MR, Mendelian randomization; IV, instrumental variable; SE, standard error. (A) Scatter plot for SAH on BD; (B) funnel plot for uIA on BD; (C) leave-one-out graph for SAH on BD; (D) forest plot for SAH on BD. [file Image_7.TIF]

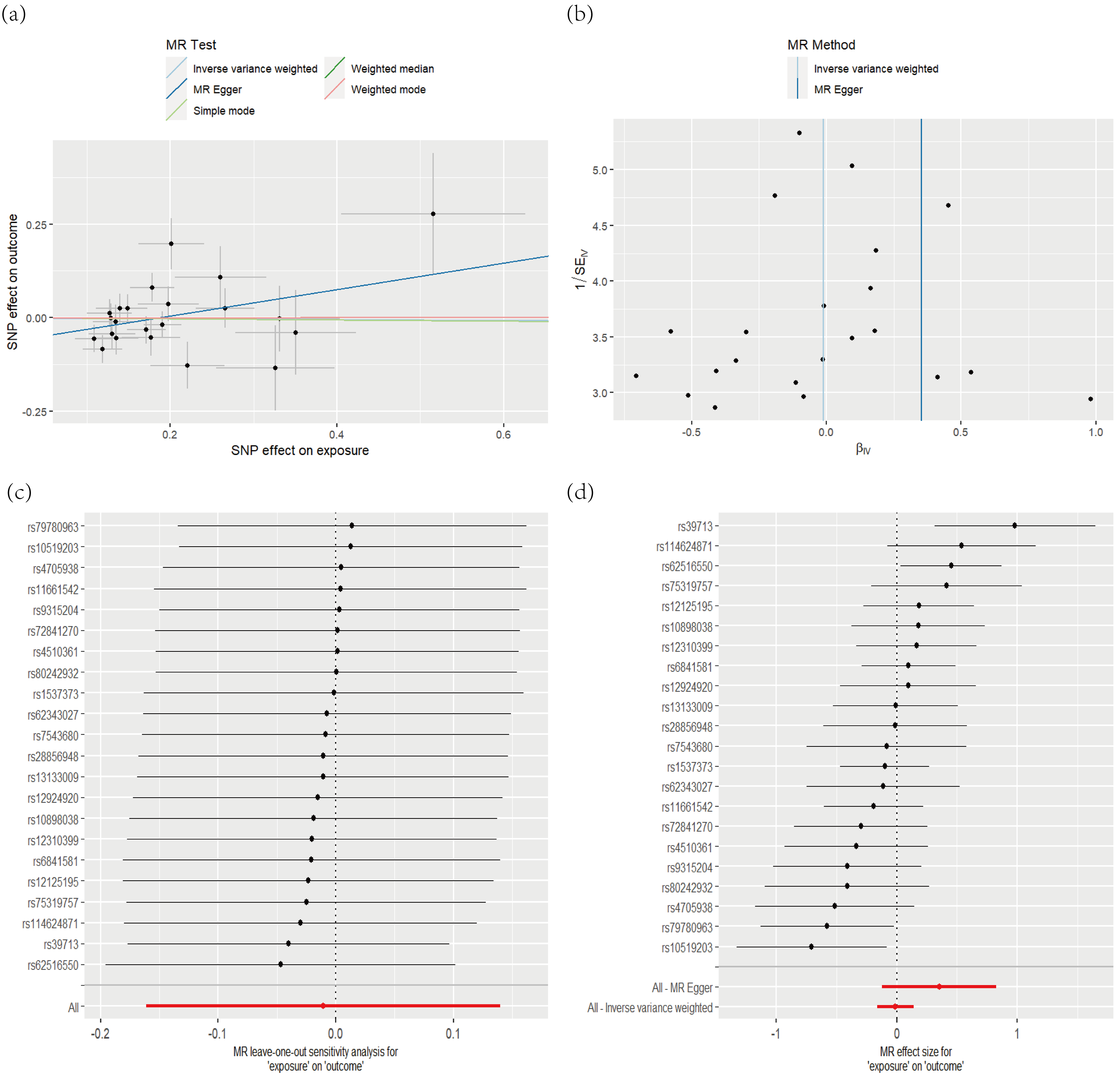

Supplement: SUPPLEMENTARY FIGURE S8 — Supplementary figures for aSAH on PD. aSAH, aneurysmal subarachnoid hemorrhage; PD, panic disorder; MR, Mendelian randomization; IV, instrumental variable; SE, standard error. (A) Scatter plot for SAH on PD; (B) funnel plot for SAH on PD; (C) leave-one-out graph for SAH on PD; (D) forest plot for SAH on PD. [file Image_8.TIF]

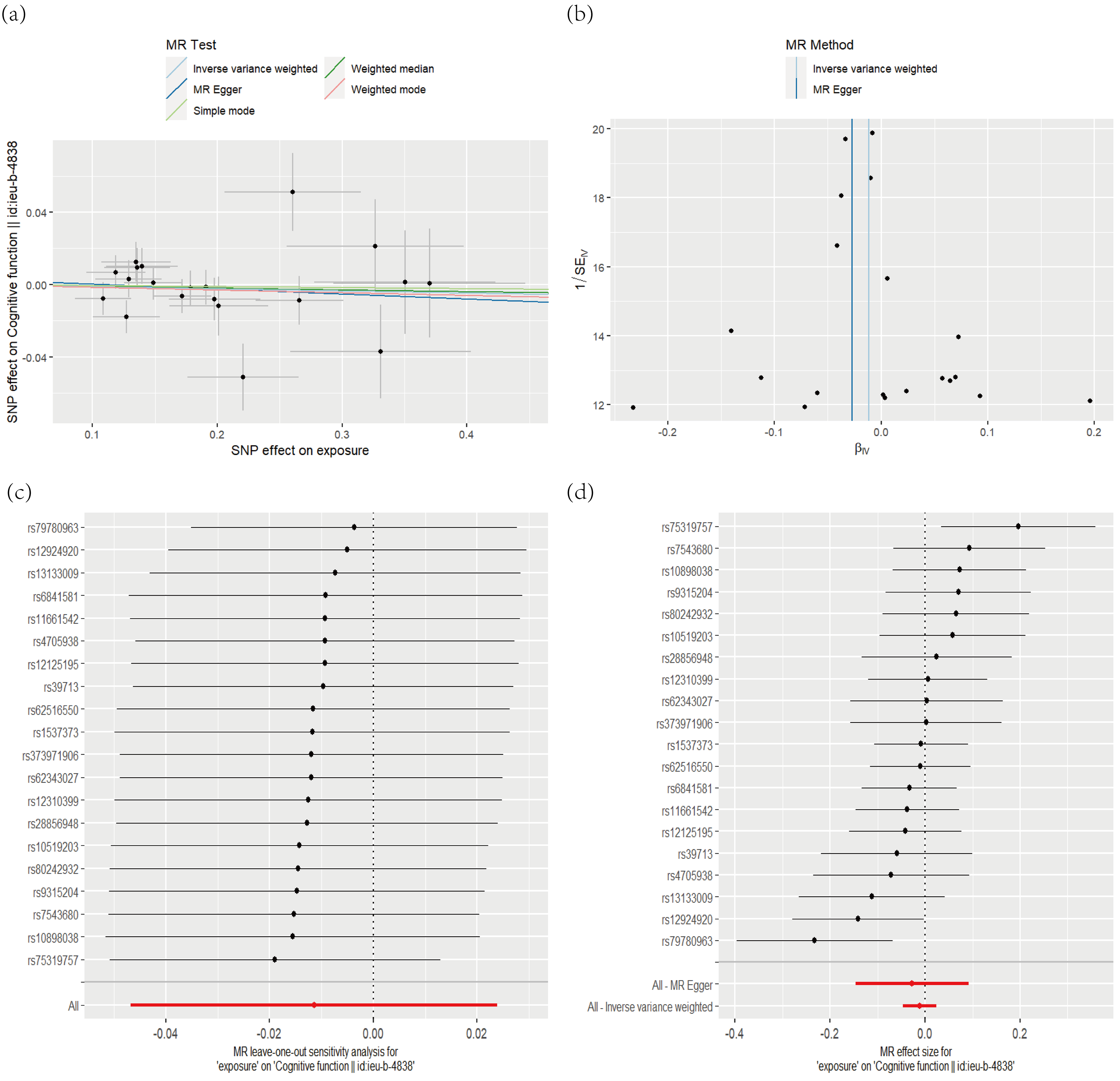

Supplement: SUPPLEMENTARY FIGURE S9 — Supplementary figures for aSAH on CF. aSAH, aneurysmal subarachnoid hemorrhage; CF, cognitive function; MR, Mendelian randomization; IV, instrumental variable; SE, standard error. (A) Scatter plot for SAH on CF; (B) funnel plot for SAH on CF; (C) leave-one-out graph for SAH on CF; (D) forest plot for SAH on CF. [file Image_9.TIF]

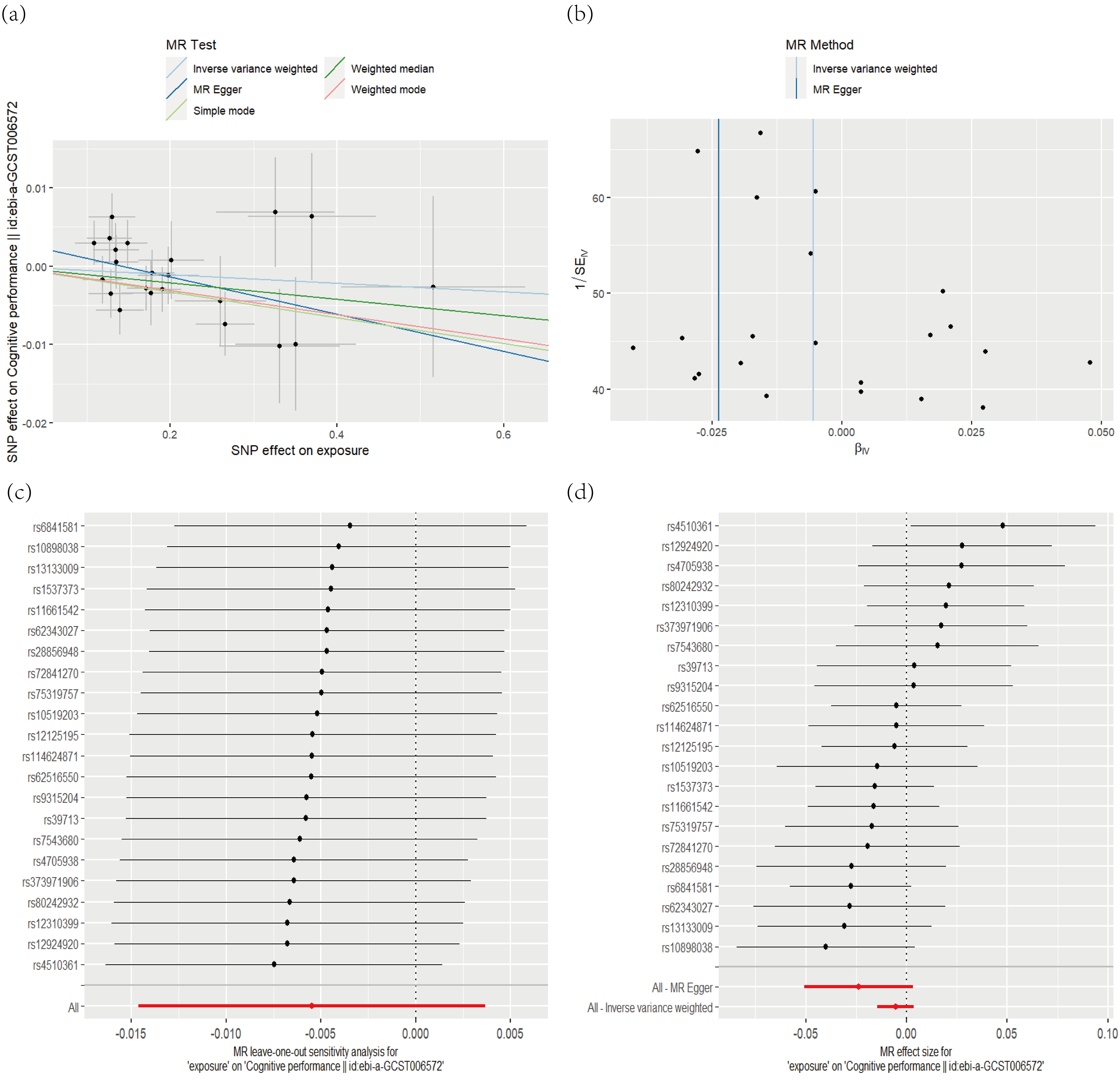

Supplement: SUPPLEMENTARY FIGURE S10 — Supplementary figures for aSAH on CP. aSAH, aneurysmal subarachnoid hemorrhage; CP, cognitive performance; MR, Mendelian randomization; IV, instrumental variable; SE, standard error. (A) Scatter plot for SAH on CP; (B) funnel plot for SAH on CP; (C) leave-one-out graph for SAH on CP; (D) forest plot for SAH on CP. [file Image_10.TIF]

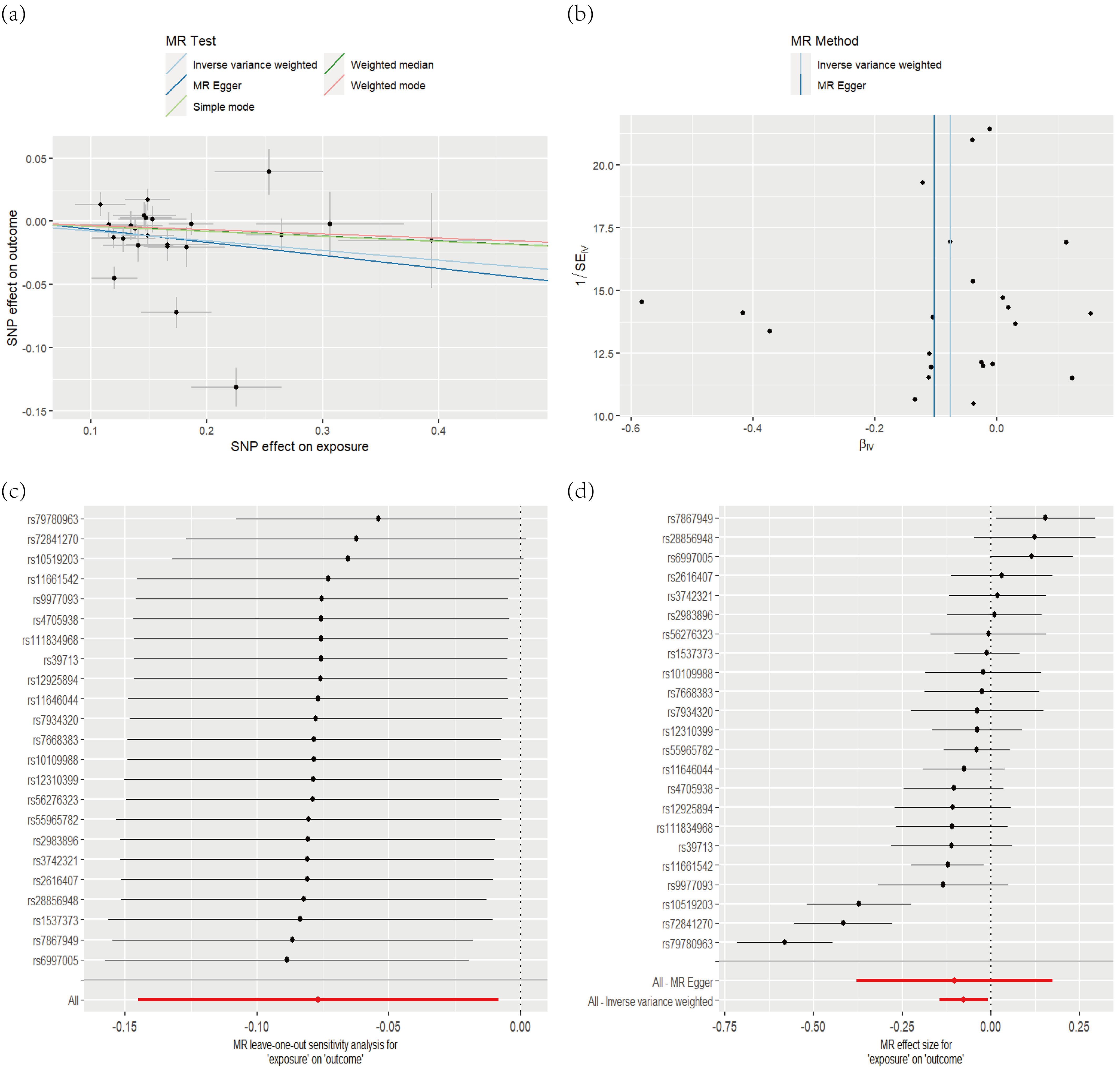

Supplement: SUPPLEMENTARY FIGURE S11 — Supplementary figures for IAs on SCZ. IAs, intracranial aneurysms; SCZ, schizophrenia; MR, Mendelian randomization; IV, instrumental variable; SE, standard error. (A) Scatter plot for IAs on SCZ; (B) funnel plot for IAs on SCZ; (C) leave-one-out graph for IAs on SCZ; (D) forest plot for IAs on SCZ. [file Image_11.TIF]

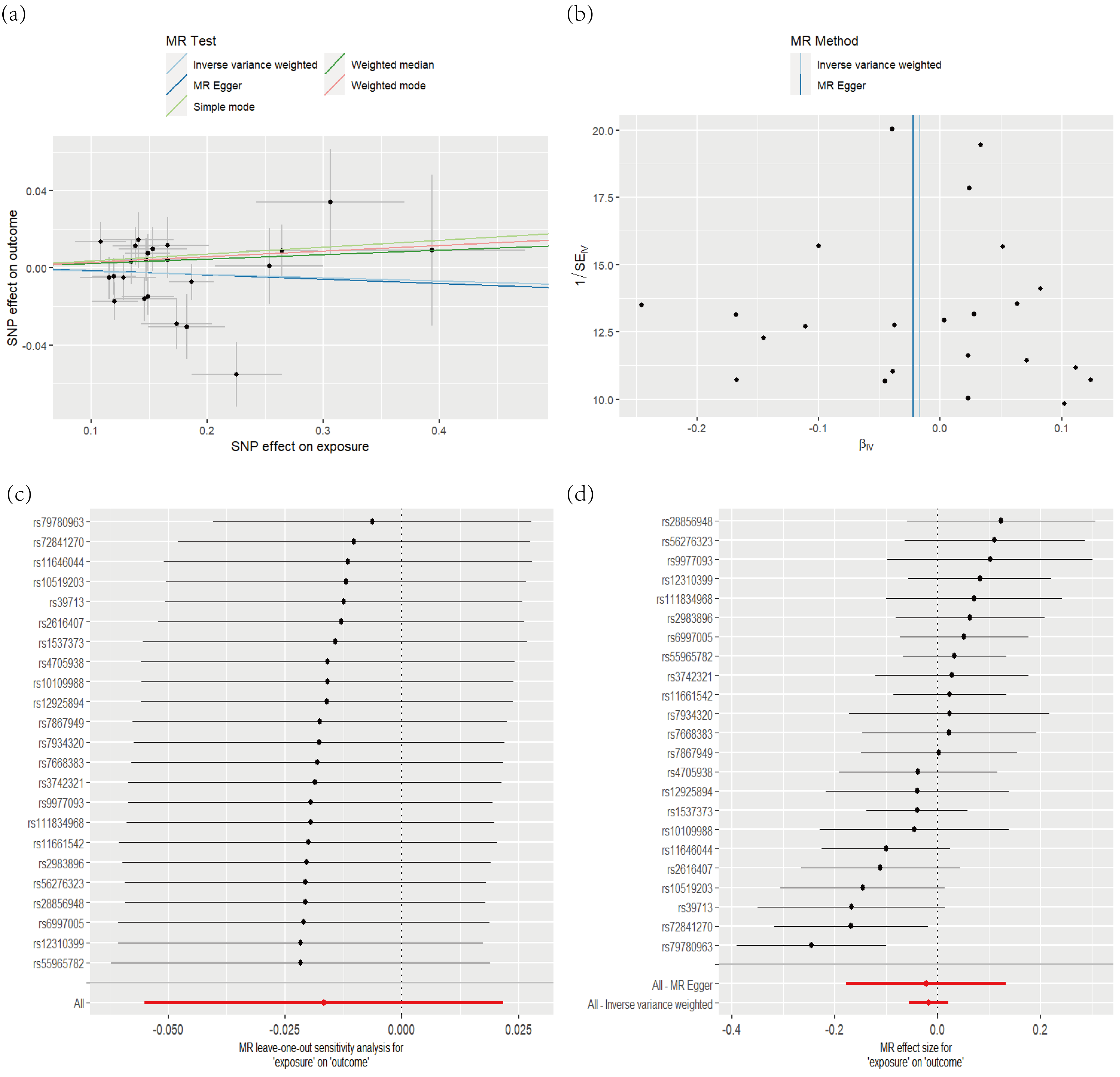

Supplement: SUPPLEMENTARY FIGURE S12 — Supplementary figures for IAs on BD. IAs, intracranial aneurysms; BD, bipolar disorder; MR, Mendelian randomization; IV, instrumental variable; SE, standard error. (A) Scatter plot for IAs on BD; (B) funnel plot for IAs on BD; (C) leave-one-out graph for IAs on BD; (D) forest plot for IAs on BD. [file Image_12.TIF]

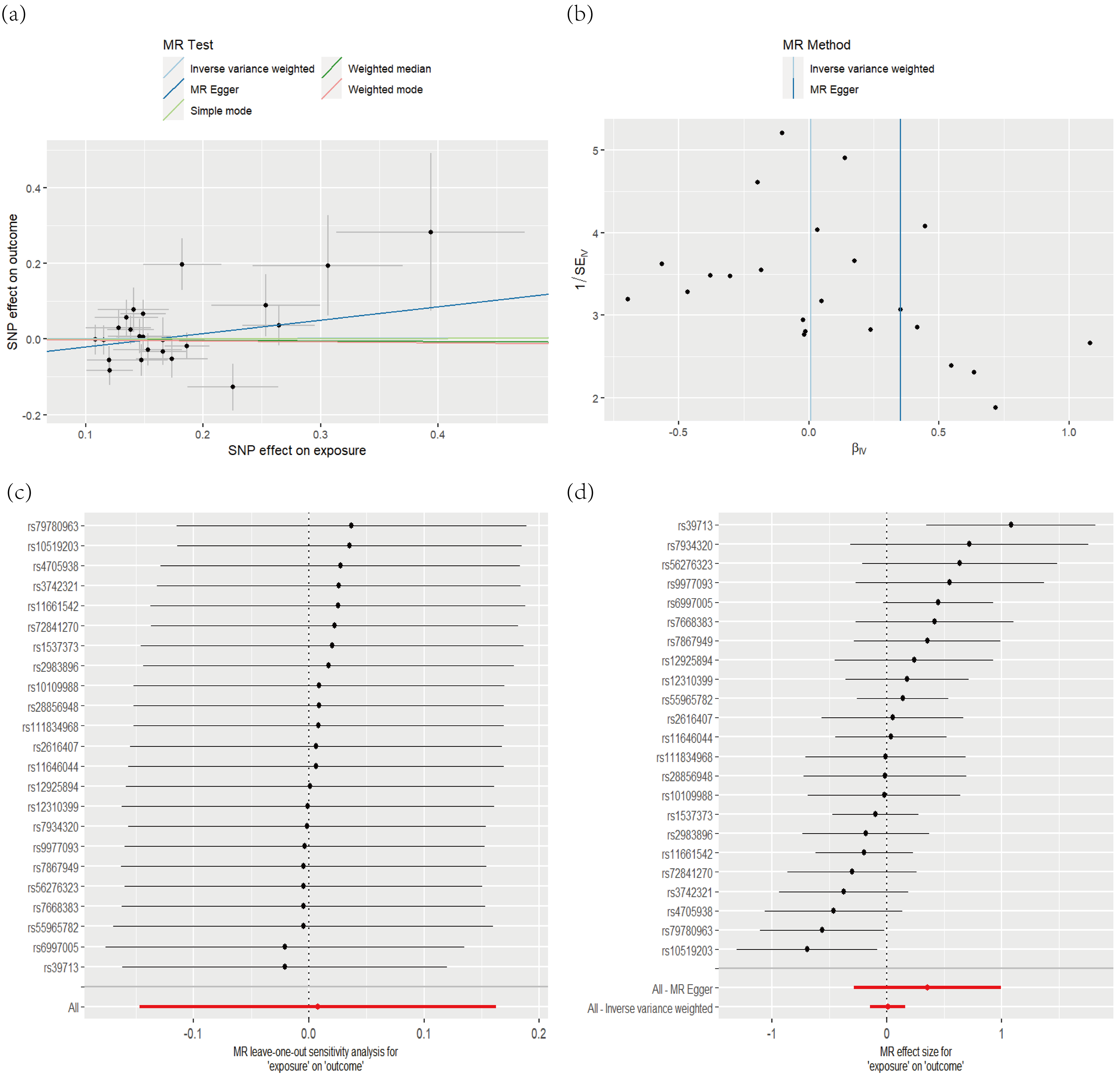

Supplement: SUPPLEMENTARY FIGURE S13 — Supplementary figures for IAs on PD. IAs, intracranial aneurysms; PD, panic disorder; MR, Mendelian randomization; IV, instrumental variable; SE, standard error. (A) Scatter plot for IAs on PD; (B) funnel plot for IAs on PD; (C) leave-one-out graph for IAs on PD; (D) forest plot for IAs on PD. [file Image_13.TIF]

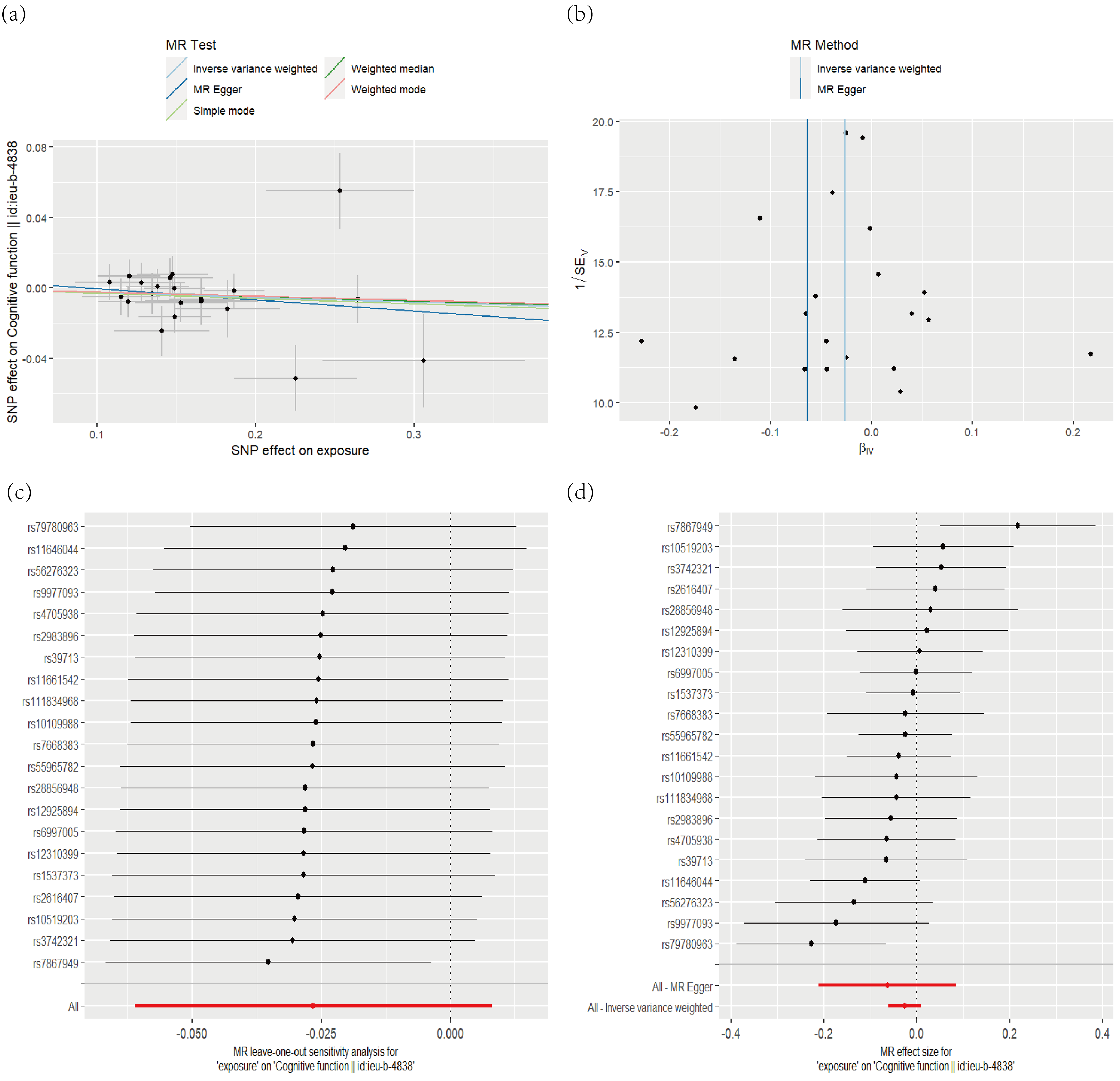

Supplement: SUPPLEMENTARY FIGURE S14 — Supplementary figures for IAs on CF. IAs, intracranial aneurysms; CF, cognitive function; MR, Mendelian randomization; IV, instrumental variable; SE, standard error. (A) Scatter plot for IAs on CF; (B) funnel plot for IAs on CF; (C) leave-one-out graph for IAs on CF; (D) forest plot for IAs on CF. [file Image_14.TIF]

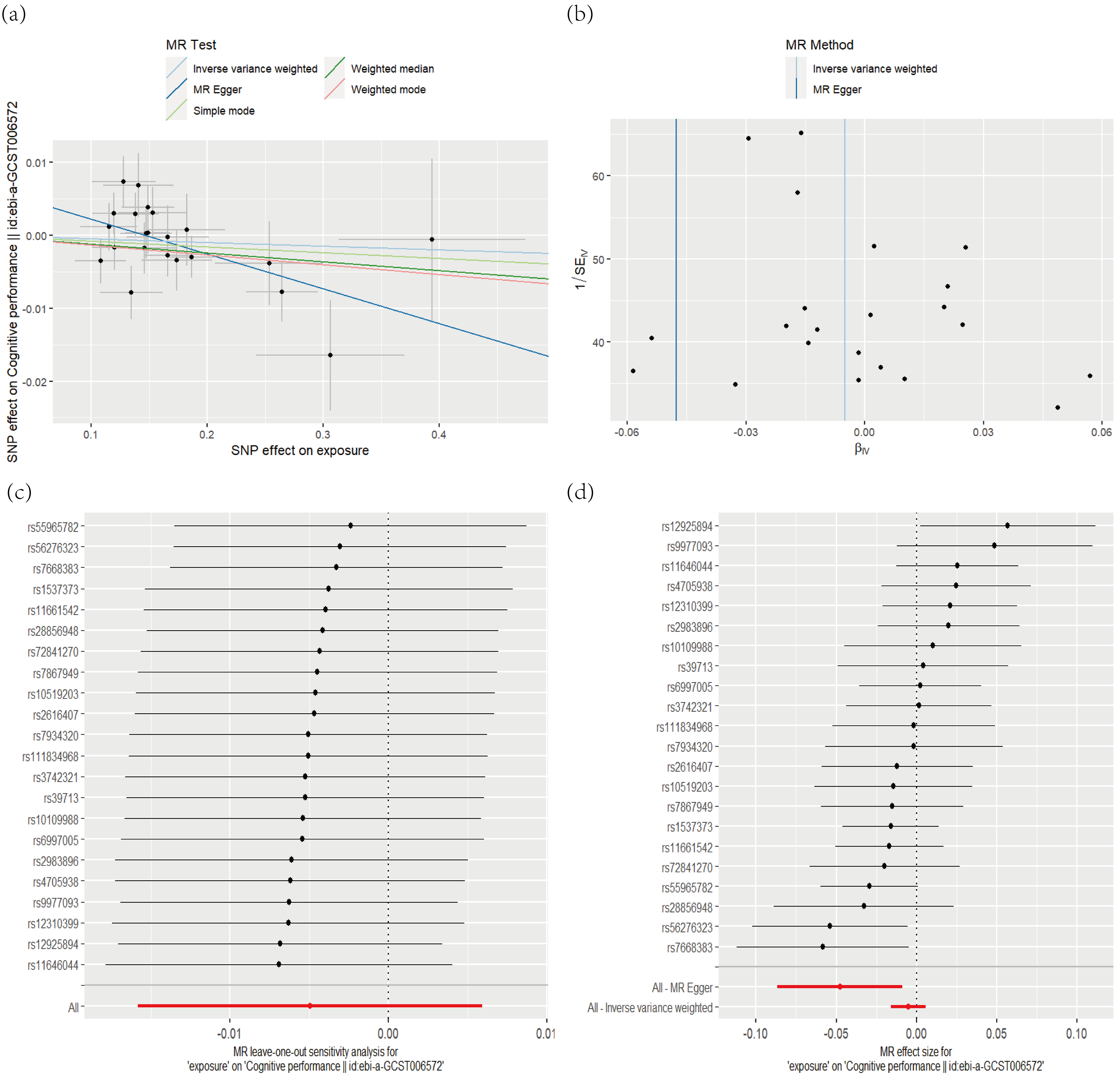

Supplement: SUPPLEMENTARY FIGURE S15 — Supplementary figures for IAs on CP. IAs, intracranial aneurysms; CP, cognitive performance; MR, Mendelian randomization; IV, instrumental variable; SE, standard error. (A) Scatter plot for IAs on CP; (B) funnel plot for IAs on CP; (C) leave-one-out graph for IAs on CP; (D) forest plot for IAs on CP. [file Image_15.TIF]
